# Sol–gel and co-precipitation synthesized hybrid nanofluids for enhanced CNC turning of AISI 4340 steel: an experimental and machine learning approach

**DOI:** 10.1038/s41598-025-25102-4

**Published:** 2025-11-21

**Authors:** Dame Alemayehu Efa, Dejene Alemayehu Ifa, Naol Dessalegn Dejene, Habtamu Zewude Belachew, Dereje Fedasa Tegegn

**Affiliations:** 1 School of Mechanical Engineering, Institute of Technology, Wallaga University, P.O. Box.395, Nekemte, Ethiopia; 2 Department of Chemistry, College of Natural and Computational Science, Dambi Dollo University, Dambi Dollo, Ethiopia; 3 Department of Chemistry, College of Natural and Computational Science, Jimma University, Jimma, Ethiopia

**Keywords:** CNC turning, AISI 4340 steel, Advanced machine learning, Nanoparticles, Mechanical engineering, Chemistry

## Abstract

Machining high-strength alloys, such as AISI 4340 steel, presents significant challenges in terms of surface integrity, production efficiency, and heat dissipation. This study investigated the effects of a novel hybrid nanofluid of copper oxide (CuO) and aluminum oxide (Al_2_O_3_) nanoparticles to improve CNC turning of AISI 4340 steel. The experiments were conducted under a range of cutting conditions by varying the cutting speed, depth of cut and feed rate, along with the concentration of the hybrid nanofluid. A new methodology for preparing and applying the hybrid nanofluid demonstrated sufficient cooling and lubrication properties, enabling machining tests that improved upon traditional methods. The experimental study indicated that as the cutting speed and feed rate increased, the cutting temperature and surface roughness also increased significantly. Increasing the nanofluid concentration (0.25–0.45%) lowered the tool tip temperature and surface roughness due to increased thermal conductivity and formation of a protective tribological film. However, beyond 0.45% hybrid nanofluid concentration, the performance declined due to increased fluid viscosity and agglomeration of nanoparticles. An Artificial Neural Network (ANN) demonstrated significant predictive accuracy, with coefficients of determination (R^2^) of 0.864 for tool tip temperature, 0.828 for surface roughness, and 0.942 for material removal rate (MRR). The Genetic Algorithm (GA) determined the optimal nanofluid concentration of 0.4%, cutting speed of 80 m/min, feed rate of 0.07 mm/rev, and depth of cut of 0.4 mm. Experimental data confirmed ANN predictions with an error range of less than ± 2%, and confirmatory trials demonstrated that heat was dissipated, showing improved surface quality and MRR.

## Introduction

The turning of AISI 4340 steel, characterized by excellent strength, toughness, and fatigue resistance, presents a major challenge in the CNC operations. Such challenges arise mainly due to the extreme hardness of the alloy, coupled with its poor machinability, resulting in very high tool-work interactions, quick tool wear, and high thermal stresses generated during cutting^1^. Conventional machining processes also suffer from setting the upper performance boundary due to inappropriate process settings and inefficiently applied coolant^2^. For example, higher setting speeds may be able to enhance productivity; however, increased thermal stress due to friction and plastic deformation will shorten tool life and reduce surface integrity^3^. High feed rates improve the removal of material but increase forces and impact cutting with vibrations and tool deflection^4^. On the other hand, lower feed rates, or very small depths of cut, ensure stability but can work against process efficiency^5^. Therefore, these interrelated parameters must be optimized sequentially for reliable and sustainable machining.

Conventional optimization methods have difficulty properly accounting for the complex relationships within problems that involve machining variables such as depth of cut, cutting speed, and feed rate^6,7^. Increasing cutting speed or feed rate increases cutting forces and cutting temperatures, and consequently rougher surface finishes and greater tool wear. Conversely, excessively low cutting speed and feed rates negatively impact productivity; therefore, a balance must be struck. Although depth of cut has received less attention compared to other variables, increasing it significantly raises the material removal rate while also increasing cutting forces, power consumption, and heat generation, which can negatively affect surface finish, dimensional accuracy, and accelerate tool wear^8,9^. Likewise, surface quality is dependent on adequate cooling/lubrication systems to help maintain cut temperatures^10^. Thus, to enhance machining performance, an overall integrated approach should be adopted, considering the numerous interrelationships involved.

Minimum Quantity Lubrication (MQL) is an alternative to flood cooling and dry machining, positioning the small quantity of lubricants supplied directly to the cutting area in aerosol droplets^11^. MQL promises both greater heat dissipation and minimum friction without the accompanying disadvantages of using conventional coolants at bulk volumes^12^. The effectiveness of MQL can be increased when enhancing it with fluids of the nanoparticle family, called nanofluids^13^. Out of the extensive range of nanoparticles explored, copper oxide (CuO) and aluminum oxide (Al_2_O_3_) showed good performance in tribology and thermal behavior^14^. Nanofluid-based MQL systems are associated with improved cooling performance, tool wear reduction, and enhanced stability of machining dynamics through lubrication and heat conduction within the cutting area^14^. The result is a more consistent output from machining, increased durability in tool life, and considerably lower fluid requirements, all while maintaining excellence in environmental space^15,16^.

An experimental study showed that CuO and Al_2_O_3_ nanoparticles-based nanofluids may reduce the cutting temperature by as much as 26% and improve their tribological characteristics, characterized by low friction and wear^17,18^. When CuO and Al_2_O_3_ are used in combination as hybrid nanofluids, their efficiency is more pronounced than that of isolated nanofluids, with considerable reductions of cutting forces and friction coefficients in high-performance machining operations^19^. The presence of these fluids helps create stable thickness lubricating films at the tool surfaces, lowers adhesion build-up, and forms chips more uniformly^20^.

Despite a nanofluid-assisted machining has demonstrated remarkable advancements, limited research has been done on hybrid CuO-Al_2_O_3_ nanofluids made by controlled techniques including sol–gel or co-precipitation to CNC turn AISI 4340 steel. This work fills this gap by developing new hybrid nanofluids with consistent particle integrity and systematically evaluating their machining capability, stability, and dispersion. Additionally, this work is distinguished by the integration of experimental investigation with advanced statistical modeling, including response surface methodology (RSM), and a hybrid metaheuristic–machine learning (ML) approach. This approach combines multiple ML techniques, such as support vector regression (SVR), gradient boosting regression (GBR), linear regression (LR), gaussian process regression (GPR), and artificial neural networks (ANN), with a genetic algorithm (GA) for accurate prediction of performance metrics and process parameters optimization.

## Materials and methods

**Fig. 1:**
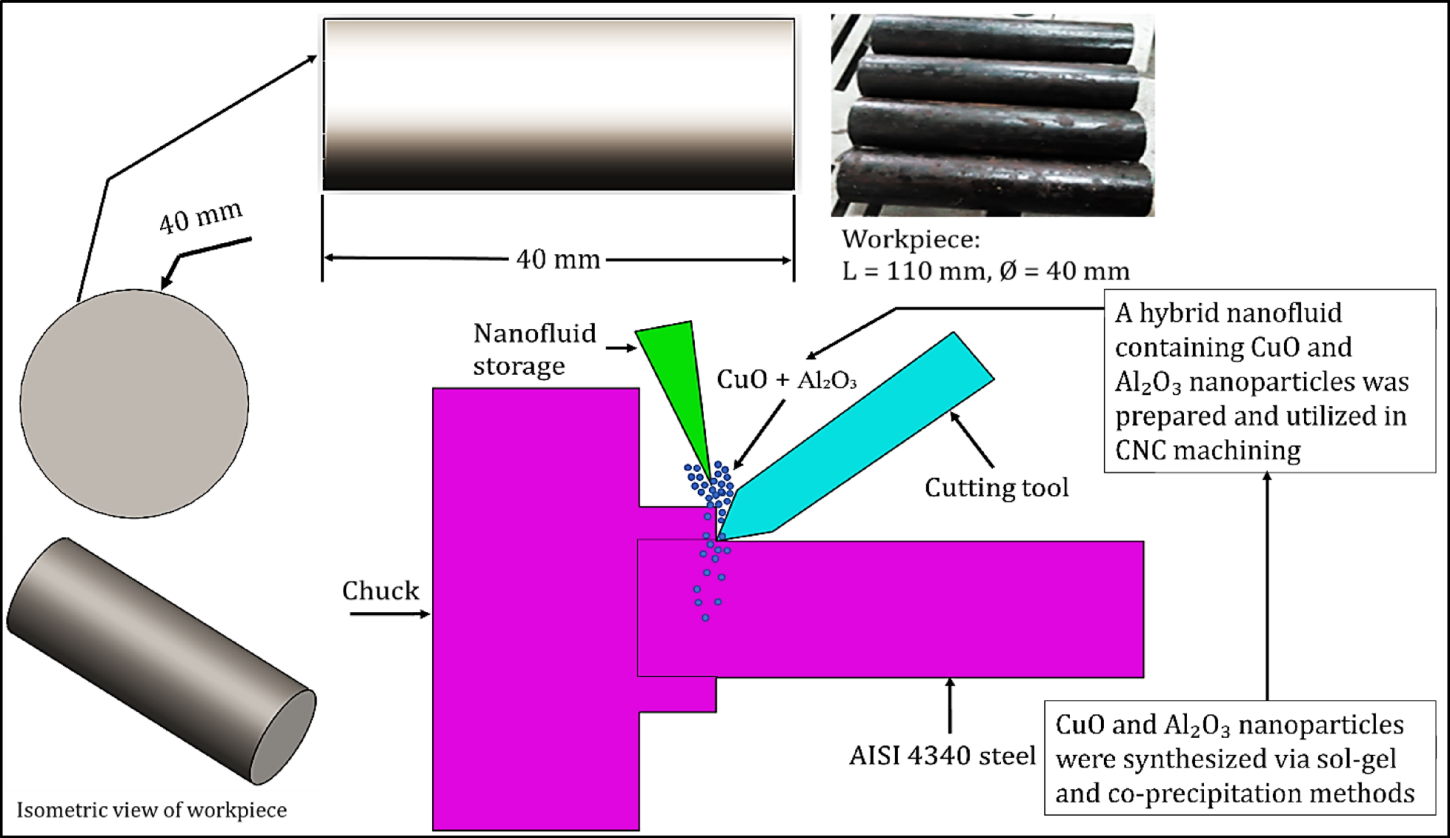
Experimental setup and workpiece dimensions.

The high strength, excellent toughness, and excellent hardenability of AISI 4340 steel^32^ are the main reasons it has been so commonly used in CNC turning applications. It also has exceptional dimensional stability and excellent fatigue resistance^21^. AISI 4340 is a suitable choice for components that require high mechanical performance and precision manufacturing^22^. It is versatile enough to be used in a variety of machining environments and suitable for a wide range of industrial applications^23^. Thus, in this study, the machining tests were performed using AISI 4340. The cylindrical sample was made to a diameter of Ø40 mm and a length of 110 mm, as shown in Fig. 1. The material was assumed to be uniform throughout the workpiece to ensure smooth cutting and minimize variability in cutting performance. The chemical properties of AISI 4340 steel are illustrated in Table 1^24^. All machining tests were conducted using a DMTG CNC lathe, model CKE 6150, which is power-fed for a 3-phase, 380 V, 50 Hz supply. It is notable for its high precision and stable turning operations on hardened steels. Cutting parameters, including spindle speed, feed rate, and depth of cut, were controlled through the CNC system. Calibration was performed for the machine before every trial to decrease systematic errors. The cutting tools used had carbide inserts (CNMG431-MA, CNMG120404), with a measurement of 12 × 5 mm (0.472 × 0.196 in) relatively. Such inserts have been selected because each is highly efficient and wear-resistant, while also being well-suited for AISI 4340 machining. They are mounted on an MCGNR-M12 tool holder, which provides rigidity and reduced vibrations during operation. Tool wear is monitored visually and assumed to be negligible for the short machining duration used. Surface roughness was measured using a VOGEL Surface Roughness Tester, model 65,711. Measurements were taken at multiple points along the machined surface, and the arithmetic average roughness (Ra) was calculated. The apparatus was calibrated before each measurement set to ensure accuracy. Tool-tip temperature was recorded using a Type K ATAL infrared thermometer with the emissivity coefficient fixed at 0.28. Measurement was done in real-time with a fixed position of the device in relation to the cutting zone. During an experiment, the emissivity condition or construction of the cutting zone was assumed to remain constant. For cooling and lubrication, Al_2_O_3_/copper hybrid nanofluids were prepared using nanoparticles synthesized via sol–gel and co-precipitation methods, and then dispersed through ultrasonication to achieve uniform stability and dispersion. The concentration of nanoparticles, sonication time, and dosage of the stabilizer were all controlled to ensure uniformity. The long-term stability of the nanofluids beyond the experimental period was not considered in this study.

**Table 1:** Chemical composition (wt%) of AISI 4340 steel.

C	Mn	S	Cr	Cu	Si	Mo	Ni	P	Fe
0.4	0.71	0.006	0.8	0.15	0.26	0.22	1.73	0.015	Bal.

### Synthesis and preparation of nanofluids

#### Synthesis of nanoparticles

The most straightforward methods for creating nanoparticles (NPs) in a range of sizes and shapes are sol–gel and co-precipitation^25^. Hexagonal and spherical particles are made by the co-precipitation method, while spherical particles are made by the sol–gel method^26^. The sol–gel method was used to create the necessary NPs, and Merck in the USA provided the ingredients needed for NP synthesis, which had a purity of > 99.9%^27–29^.

##### ***Al***_***2***_***O***_***3***_*** nanoparticle synthesis***

**Fig. 2:**
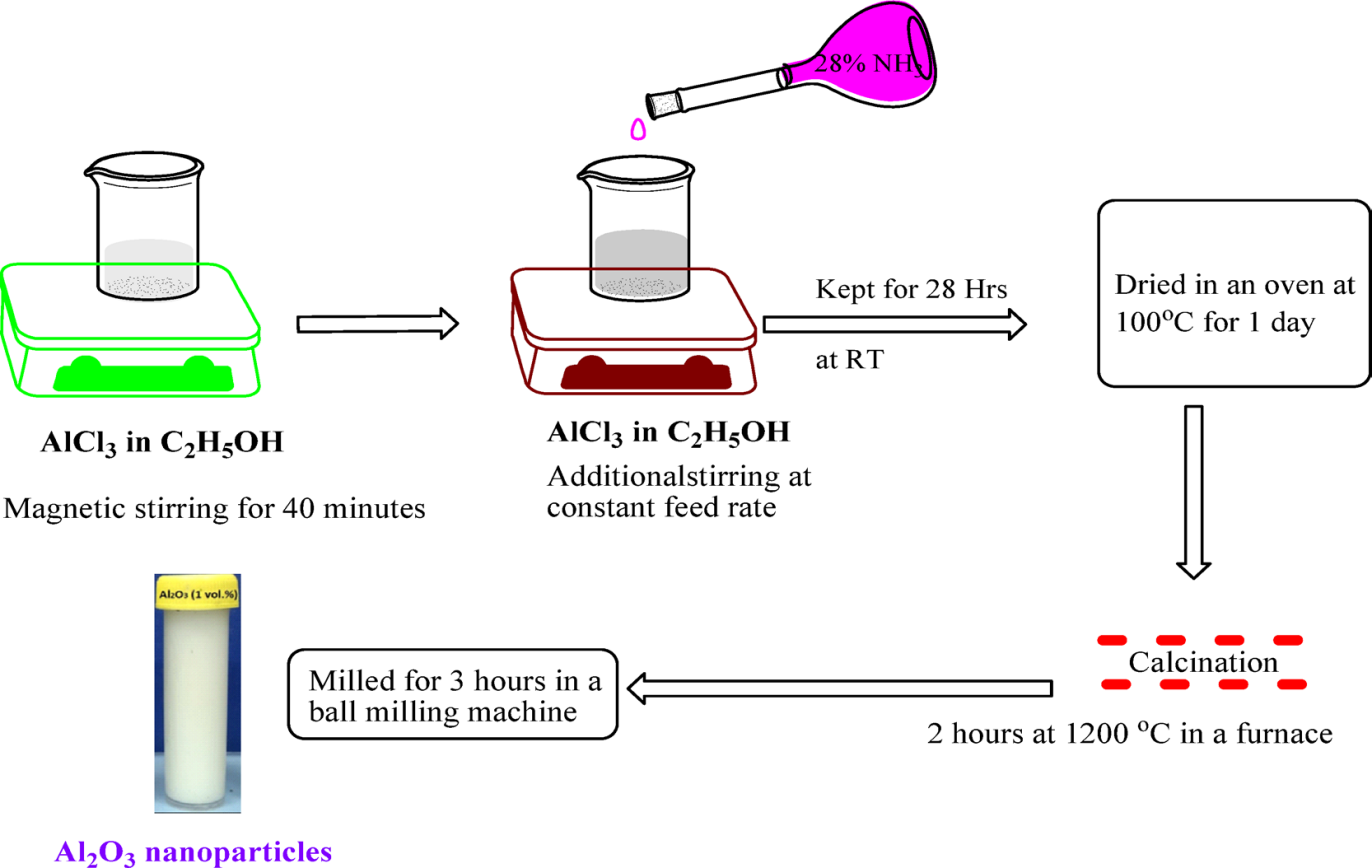
Schematic diagram of Al_2_O_3_ nanoparticle synthesis process.

The precipitating agent was ammonia (NH3) at a 28% concentration, and the precursor solution was aluminum chloride (AlCl_3_) in ethanol. A 0.1 M ethanolic solution of AlCl_3_ was magnetically stirred for 40 min, and the controlled addition of NH_3_ resulted in the formation of a gel. The gel was aged at room temperature for 28 h, oven-dried at 100 °C for 24 h, and further calcined at 1200 °C for 2 h in a furnace^30^. The calcined powder was ball-milled (Model: Retch, PM-100) for 3 h to obtain nanosized α-Al_2_O_3_ particles. The flow chart of the process is given in Fig. 2.

##### CuO nanoparticle synthesis

**Fig. 3:**
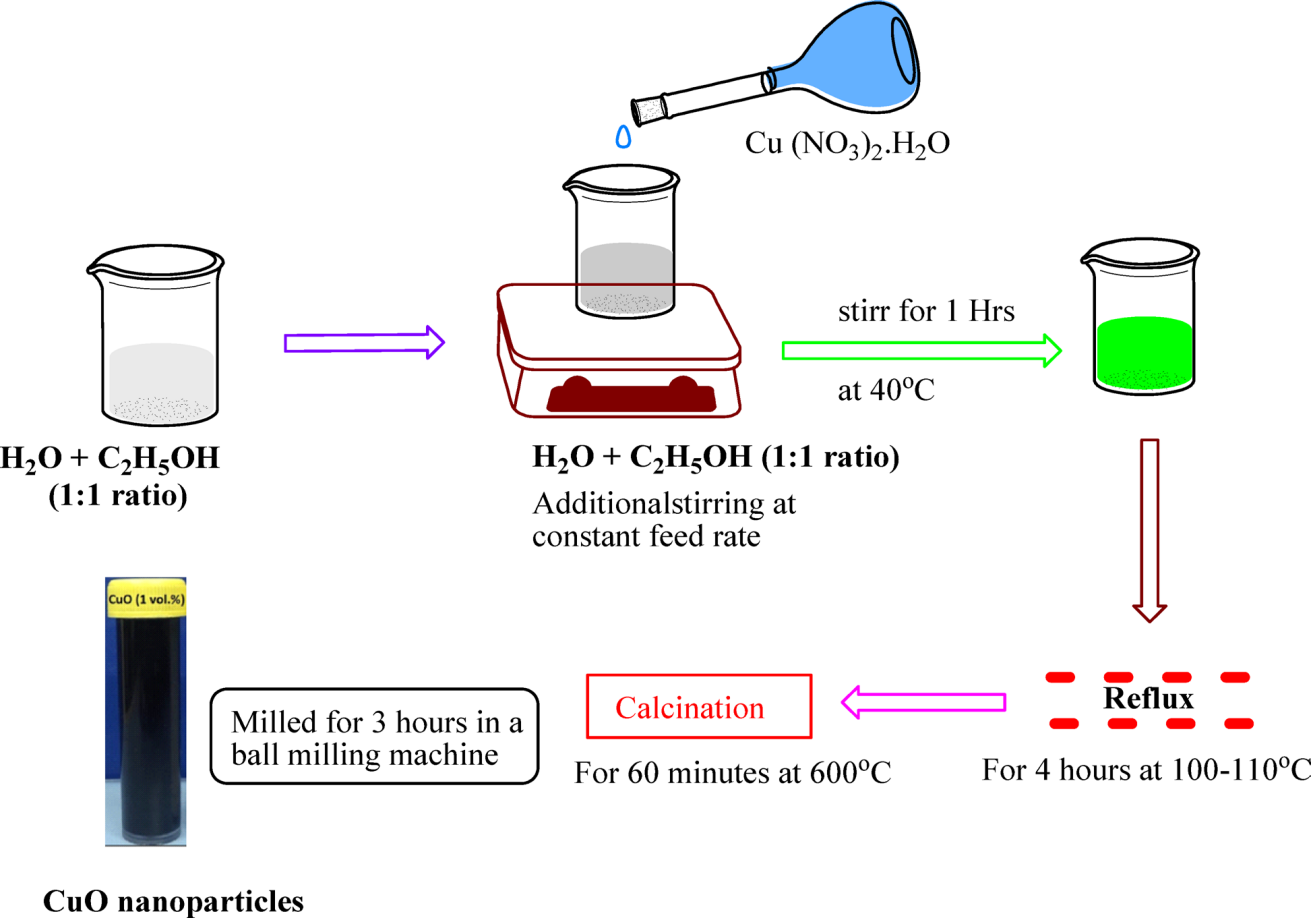
Schematic diagram of CuO nanoparticle synthesis process.

A precursor solution was prepared using a 1:1 ratio of distilled water and ethanol. Copper nitrate [Cu (NO_3_)_2_.H_2_O] was then added. A green solution was developed at 40 °C after an hour of stirring. For four hours, the homogenous mixture was kept in reflux at 100–110 °C. The excess solvents were vaporized to produce a moist gel. After being calcined for 60 min at 600 °C, the black gel powder was ball-milled for three hours^31^. Citric acid and EG were used as complexing and polymerization agents, and the entire synthesis process is illustrated in Fig. 3.

#### ***Al***_***2***_***O***_***3***_***-CuO nanofluids preparation***

The preparation of Al_2_O_3_-CuO nanofluid involves dispersing alumina (Al_2_O_3_) and copper oxide (CuO) nanoparticles in a base fluid (distilled water). The most popular method for NF preparation is the two-step procedure^32^. Al_2_O_3_ nanoparticles were initially suspended and dispersed independently before being combined with an equal proportion of CuO (50% Al_2_O_3_ + 50% CuO) in base fluid (distilled water). This balanced composition at varying concentrations produced optimal enhancements in both heat transfer and tribological performance^33^. In order to stabilize the dispersion and sustain long-term stability of nanofluids, sodium dodecylbenzene sulfonate (SDBS) was used as a surfactant^34^. For the Al_2_O_3_ nanofluid, SDBS was added to the base fluid at 0.2 times the nanoparticle weight, while it was introduced at 0.4 times the nanoparticle weight for the hybrid nanofluid, with subsequent vigorous stirring of the 60 mL base liquid before addition of the nanoparticles.

**Fig. 4:**
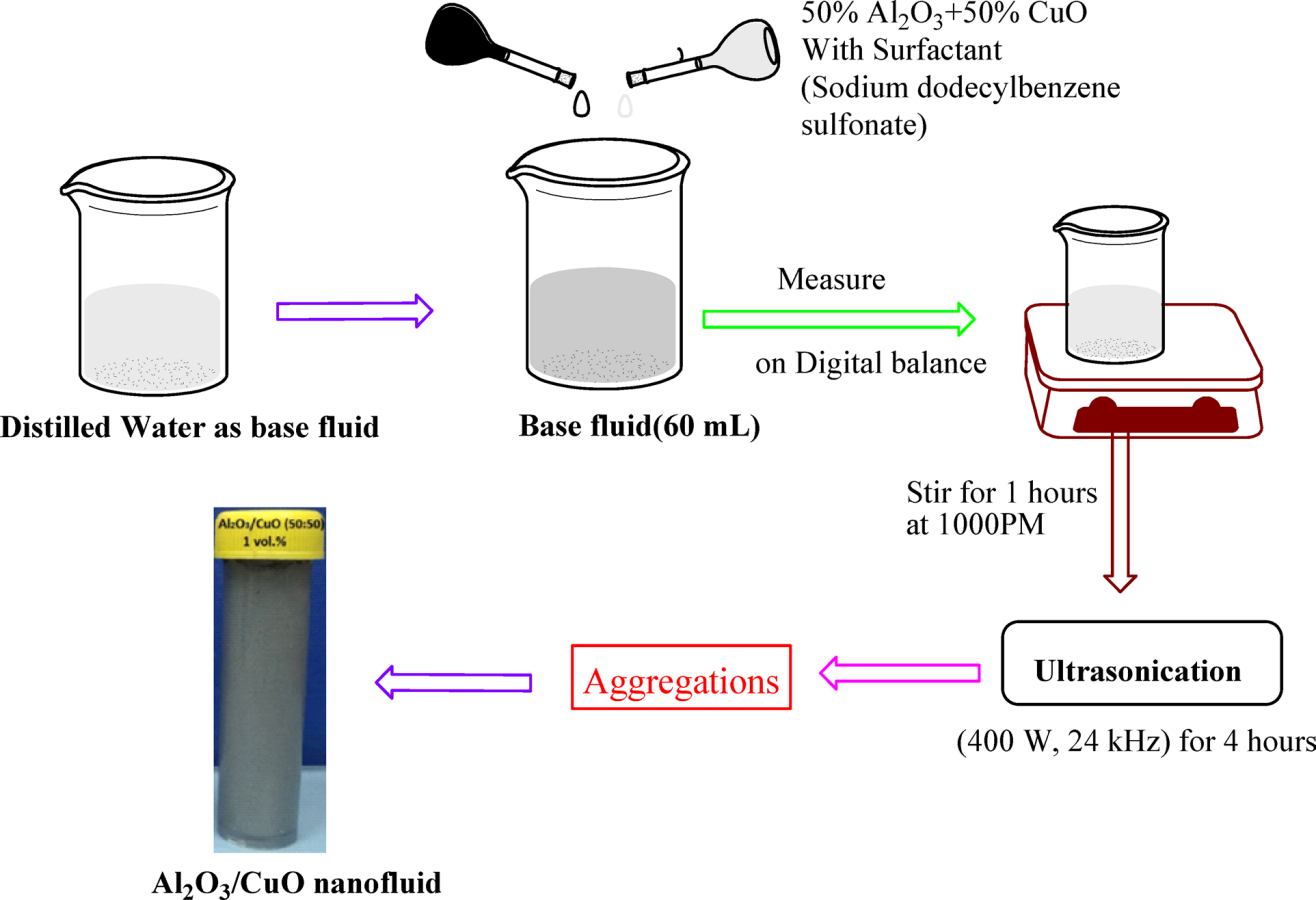
Schematic diagram of Al_2_O_3_-CuO nanofluid synthesis process.

**Fig. 5:**
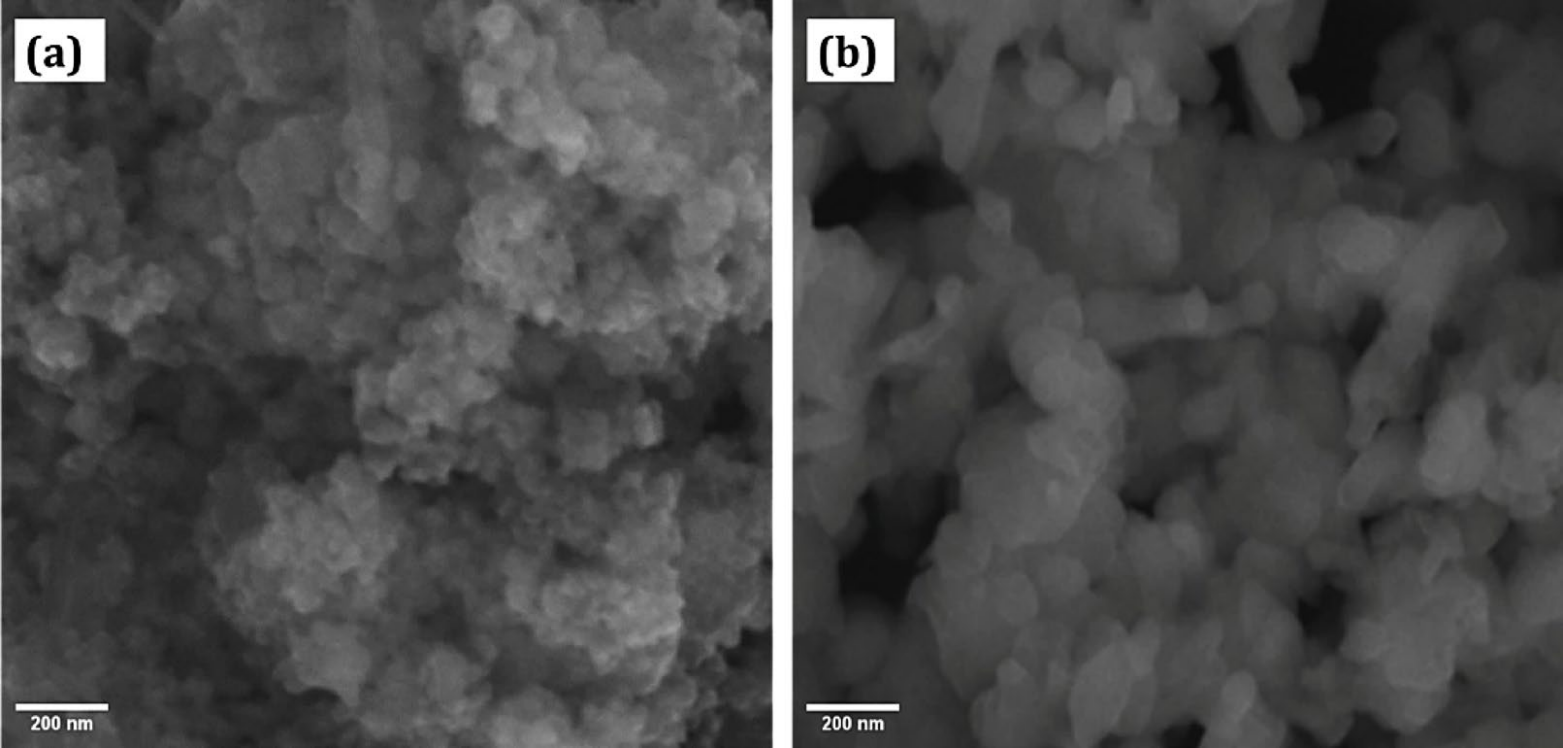
SEM images of (**a**) Al_2_O_3_ and (**b**) CuO.

The estimated quantity of NPs was measured using a digital balance (Wenar, PGB-3010) and then distributed in distilled water. After that, the suspension is agitated for one hour at 1000 PM using a magnetic stirrer (REMI 2MLH). The concentrations were then subjected to four hours of ultrasonication (400 W, 24 kHz). Particle aggregation was broken down using this method, resulting in a uniform dispersion and suspension^35^. As shown in Fig. 4, this process resulted in a stable and uniform hybrid nanofluid. The physical characteristics of the synthesized nanoparticles, such as their sizes, densities, specific temperatures, and thermal conductivities, are listed in Table 2. Figure 5 displays representative SEM images of CuO and Al_2_O_3_ nanoparticles.

**Table 2:** Characteristics of nanoparticles used in this research.

Specifications	Al_2_O_3_	CuO
Average particle size (nm)	30	13
k, (W/m.K)	36	76.5
⍴, (kg/m^3^)	3970	6300
Particle shape	Spherical	Spherical
C_p_, (J/kg.K)	773	535

To express the concentration of nanoparticles in the fluid, volume concentration is typically used. This parameter represents the ratio of the volume of nanoparticles to the total volume of the nanofluid. The volume concentration of hybrid nanoparticles used in this study was calculated using a standard mathematical formula (Eq. 1)^36,37^.

1 $${C}_{nf} = \left[\frac{\left(\frac{{m}_{CuO}}{{\uprho }_{CuO}}\right)+\left(\frac{{m}_{Al2O3}}{{\uprho }_{Al2O3}}\right)}{\left(\frac{{m}_{CuO}}{{\uprho }_{CuO}}\right)+\left(\frac{{m}_{Al2O3}}{{\uprho }_{Al2O3}}\right)+\text{V}}\right]\times 100$$

#### Characterization of nanofluid properties

##### Thermophysical properties

Effective density and specific heat of hybrid nanofluids are usually assessed according to some weighted average models^38^. These parameters are mainly used to evaluate the possible convective heat transfer of the fluid. Thermal conductivity is widely modeled through correlations such as those of Maxwell and Hamilton–Crosser, although mostly direct measurements using either the KD2 Pro or transient hot-wire methods are also used. Consistent results have indicated that hybrid nanofluids enhance conductivity by about 10–20% at a low particle load, which generally is at or below 1 vol%^39,40^. The viscosity determined using standard viscometers like the Brookfield often shows slight increases with the addition of nanoparticles. Although this condition exhibits shear resistance, it stabilizes the lubricating film, thereby enhancing tribological performance without significantly impairing flowability during machining^41^.

##### Tribological, wettability, and stability properties

Tribological performance of hybrid nanofluids is thoroughly investigated using pin-on-disc tribometry with steel counter-faces, and results reveal that the coefficient of friction is lower than that of the base fluid^42^. This behavior, in fact, assists in reducing tool–chip interface friction within the machining environment. Wettability, which is measured with goniometric measurements, is another parameter that showcases the benefits of hybrid formulations. The reduced contact angles compared to the base fluid allow for enhanced spreading across the workpiece surface, therefore providing superior cooling and lubrication^43^. Zeta potential (stability) is equally important for long-term applications and has been confirmed through studies using dynamic light scattering, wherein zeta potential values higher than ± 30 mV correspond to a strong electrostatic stabilization, preventing any agglomeration, thus ensuring that the nanofluid will perform uniformly in time^44^.

##### Relevance to machining

In combining them, the improved properties of thermal conductivity and specific heat, moderated viscosity, enhanced wettability, and enhanced colloidal stability give the complete machining performance. It enhances heat management, reduces friction at the tool–chip interface, and reduces surface roughness. The CNC turning of AISI 4340 steel benefited greatly from this resistivity, with numerous studies reporting that improvements in tool life and measurable enhancements in surface finish were statistically significant compared to both base fluids and single-component nanofluids^45,46^.

### Machine learning model

The four machine learning models used to characterize the complex nonlinear characteristics of the machining response were SVR, GPR, ANN, and LR. The selection of SVR was due to its robustness against overfitting and its capability in high-dimensional spaces with limited datasets. GPR was preferred due to its probabilistic predictions and associated confidence intervals, which help us understand forecast uncertainty^47^. ANN, exploiting its multilayer structure and adaptive learning, was utilized to capture the highly nonlinear interactions among machining parameters. Meanwhile, LR was included to facilitate a comparison with improvements in performance over widely used traditional models^36^. All models were trained on RSM-generated experiments with internal validation via cross-validation and external validation using the error metrics of R^2^, MSE, and RMSE. A total of 60 experiments were performed to provide enough data to train reliable machine learning models. This approach has the potential to improve the robustness and statistical reliability of the experimental data.

### Experimental design

The input parameters for CNC turning of AISI 4340 steel, as presented in Table 3, were selected based on previous studies^2,48–50^. In this work, a Central Composite Design (CCD) within the framework of Response Surface Methodology (RSM) was employed using Design-Expert® version 13 software to facilitate optimization.

**Table 3:** Input parameters.

Input parameters	Cutting speed		Feed rate	Depth of cut	Nanofluid concentration
Symbol	${V}_{c}$		$f$	${a}_{p}$	${C}_{nf}$
Unit	m/min		mm/rev	mm	%
Level	− α	80	0.05	0.4	0.250
	0	140	0.10	0.6	0.375
	+ α	200	0.15	0.8	0.500

## Results and discussion

**Fig. 6:**
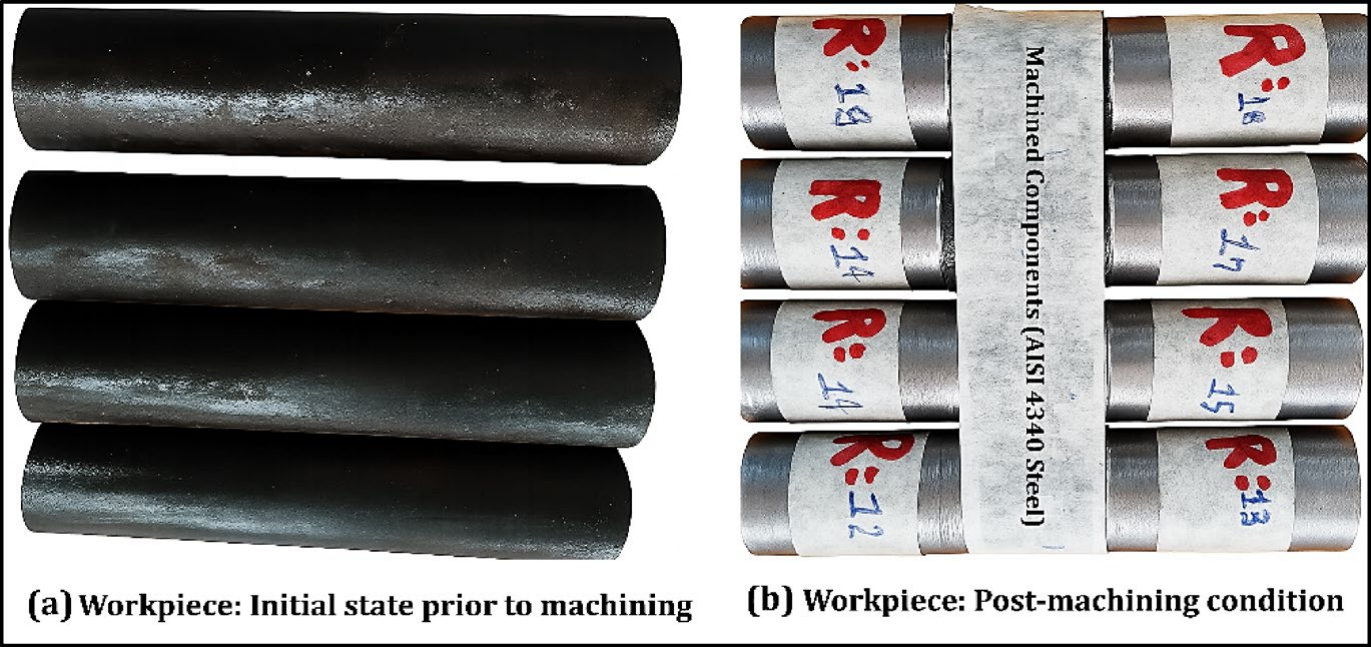
Sample illustration of workpiece pre- and post-machining.

This section presents and discusses the results obtained from the CNC turning experiments conducted on AISI 4340 steel using CuO and Al_2_O_3_ hybrid nanofluids as cutting fluids. A representative illustration of the workpiece before and after machining is shown in Fig. 6, highlighting the changes in surface characteristics due to the machining process. The complete set of experimental results is provided in Table 4, which details the observed responses under various combinations of process parameters. These responses include tool tip temperature (${T}_{tp}$), surface roughness (Ra) and material removal rate (MRR), key indicators of machining performance.

**Table 4:** The experimental results with inputs and responses.

Run	${V}_{c}$	$f$	${a}_{p}$	${C}_{nf}$	${T}_{tp}$	Ra	MRR
	m/min	mm/rev	mm	%	$^\circ{\rm C}$	µm	mm^3^/min
1	140	0.1	0.6	0.375	35.0	1.19	8400
2	140	0.1	0.6	0.375	35.0	1.19	8400
3	140	0.1	0.6	0.375	35.0	1.19	8400
4	200	0.15	0.8	0.250	40.0	1.46	24,000
5	200	0.15	0.8	0.500	42.0	1.41	24,000
6	140	0.1	0.6	0.250	36.0	1.25	8400
7	80	0.15	0.4	0.500	37.0	1.13	4800
8	80	0.1	0.6	0.375	34.0	1.14	4800
9	140	0.1	0.8	0.375	36.5	1.18	11,200
10	80	0.15	0.8	0.250	37.5	1.25	9600
11	80	0.05	0.4	0.500	29.0	1.15	1600
12	200	0.05	0.8	0.250	38.5	1.33	8000
13	140	0.15	0.6	0.375	37.5	1.29	12,600
14	200	0.15	0.4	0.250	39.0	1.39	12,000
15	140	0.1	0.6	0.500	37.0	1.34	8400
16	80	0.15	0.8	0.500	39.0	1.28	9600
17	80	0.05	0.8	0.250	34.0	1.27	3200
18	200	0.15	0.4	0.500	37.0	1.31	12,000
19	200	0.05	0.8	0.500	35.5	1.30	8000
20	200	0.1	0.6	0.375	37.0	1.29	12,000
21	200	0.05	0.4	0.250	35.5	1.34	4000
22	200	0.05	0.4	0.500	33.0	1.30	4000
23	140	0.1	0.6	0.375	35.0	1.27	8400
24	140	0.05	0.6	0.375	35.5	1.25	4200
25	80	0.15	0.4	0.250	35.0	1.27	4800
26	80	0.05	0.8	0.500	36.5	1.18	3200
27	80	0.05	0.4	0.250	29.0	1.29	1600
28	140	0.1	0.4	0.375	35.0	1.24	5600
29	140	0.1	0.6	0.375	35.0	1.19	8400
30	140	0.1	0.6	0.375	35.0	1.19	8400

### Analysis of variance (ANOVA)

The ANOVA results for tool tip temperature, surface roughness, and material removal rate are presented in Table 5, along with the sum of squares (SS) and the contribution percentage (CP) of each input parameter to the respective responses. These results help to identify the most influential factors affecting each machining outcome. Model selection was conducted using a sequential sum of squares, for which the level of significance was at 5% (P < 0.05). Furthermore, the predictive regression equations for tool tip temperature, surface roughness, and material removal rate are provided in Eqs. (2), (3), and (4), respectively.

2 $$\begin{aligned} T_{tp} = & {21}.{21} + 0.117V_{c} - 30.14C_{nf} + 15.95a_{p} + 9.41f \\ & - 0.095V_{c} \times C_{nf} - 0.028V_{c} \times a_{p} - 0.093V_{c} \times f \\ & + 13.7C_{nf} \times a_{p} + 65C_{nf} \times f - 46.87a_{p} \times f \\ & - 0.0001V_{c}^{2} + 39.29C_{nf}^{2} - 3.39a_{p}^{2} + 245.61f^{2} \\ \end{aligned}$$

3 $$\begin{aligned} Ra = & {2}.0{15} + 0.0017V_{c} - 4.01C_{nf} + 0.168a_{p} - 5.02f \\ & + 0.0011V_{c} \times C_{nf} + 0.0001V_{c} \times a_{p} + 0.0054V_{c} \times f \\ & + 0.65C_{nf} \times a_{p} + 0.6C_{nf} \times f + 1.87a_{p} \times f \\ & - 3.67{\text{E}} - 06V_{c}^{2} + 39.29C_{nf}^{2} - 0.456a_{p}^{2} + 16.701f^{2} \\ \end{aligned}$$

4 $$\begin{aligned} MRR = & {84}00 - 60V_{c} + 3.4{\text{E}} - 11C_{nf} - 14000a_{p} \\ & - 84000f - 1.226{\text{E}} - 13V_{c} \times C_{nf} + 100V_{c} \times a_{p} \\ & + 600V_{c} \times f + 3.63{\text{E}} - 11C_{nf} \times a_{p} + 3.27{\text{E}} - 10C_{nf} \times f \\ & + 1.4{\text{E}} + 05a_{p} \times f - 2.81{\text{E}} - 15V_{c}^{2} + 39.29C_{nf}^{2} \\ & + 3.37{\text{E}} - 9.94{\text{E}} - 11a_{p}^{2} - 1.38{\text{E}} - 10f^{2} \\ \end{aligned}$$

**Table 5:** ANOVA results.

Parameter	${T}_{tp}$			Ra			MRR		
	SS	P-value	CP	SS	P-value	CP	SS	P-value	CP
${V}_{c}$	39.01	< 0.0001	20.40	0.0761	< 0.0001	40.58	${2.333 x 10}^{8}$	< 0.0001	29.02
$f$	78.12	< 0.0001	40.86	0.0080	0.0603	4.266	${3.175 x 10}^{8}$	< 0.0001	39.49
${a}_{p}$	50.00	< 0.0001	26.15	0.0032	0.2189	1.706	${1.411 x 10}^{8}$	< 0.0001	17.55
${C}_{nf}$	0.1250	0.7465	0.065	0.0113	0.0295	6.026	0.0000	1.0000	0.000
Residual	17.30			0.0291			${5.760 x 10}^{6}$		
Total	191.17			0.1875			${8.039 x 10}^{8}$		

### Interaction analysis

In this section presents the interactions analysis for the independent variables and dependent variables. The interaction analysis is done using response surface plots and contour diagrams. These graphical tools are based on a polynomial model that reveals interactions between two variables at a time, while the other variables are fixed at their median levels.

#### Tool tip temperature

##### Cutting speed

**Fig. 7:**
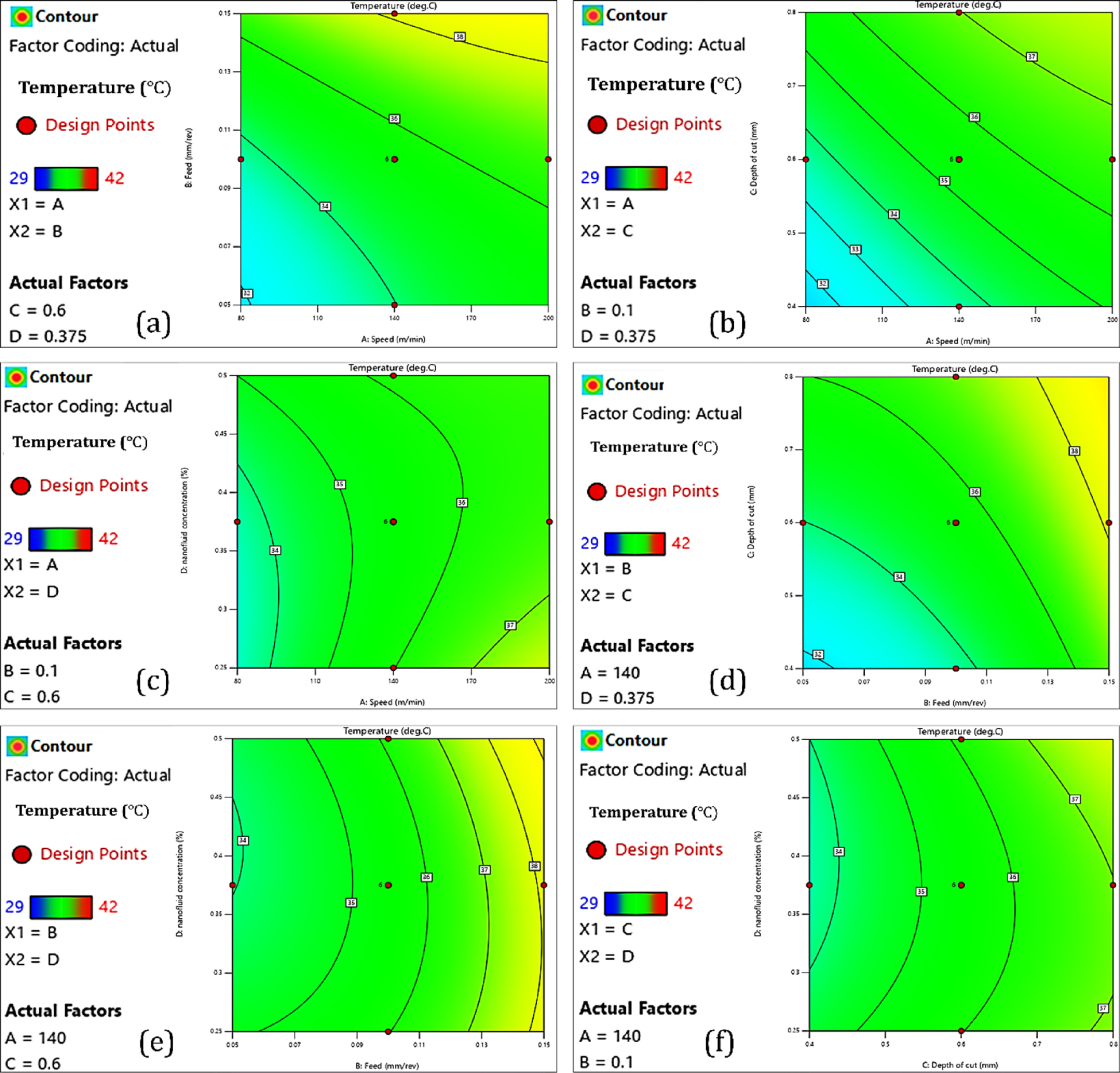
Interaction analyses of input parameters with cutting temperature.

The interaction analysis of cutting speed, feed rate, depth of cut, and nanofluid concentration on cutting temperature is illustrated in Fig. 7. In addition, Fig. 10a-a3 displays the interaction effects of the input parameters on cutting temperature. In the CNC turning process of AISI 4340 steel, increasing the cutting speed from 80 to 200 m/min resulted in drastic increases in tool temperature, as can be seen in Fig. 7a, b and c. The increase in tool temperature primarily results from the heat generated by various sources during the machining process. The heat is generated from plastic deformation occurring in the primary shear zone^51^, friction between the chip and tool rake face (secondary heat source)^52^, and friction at the tool–workpiece interface (tertiary heat source)^53^. The intensity of friction at the tool–chip interface is the main source of heat generation in the process, which is also influenced by the properties of the material being cut^54^. As cutting speed increases, so does the rate of strain in the shear zone, leading to greater amounts of plastic deformation and therefore more heat generation^55^. Concurrently, the cutting tool moves more quickly across the surface of the workpiece, thus reducing the time available for heat to dissipate. This lack of dissipated heat, in turn, leads to heat buildup at the tool–chip interface, significantly increasing the localized temperature of the cutting tool. Furthermore, the rapid speed of the machining process does not allow enough time for heat to be effectively transferred into the workpiece, chips, or the surrounding environment. Much of the heat remains near the cutting zone with little loss being distributed into the chip’s environment, which further increases thermal loading on the tool.

##### Feed rate

The effect of increasing the feed from 0.05 to 0.15 mm/rev is significant concerning tool tip temperatures, as shown in Fig. 7a, d and e. Greater feed rate increases the MRR, which means that much more material is sheared off within a given timeframe^56^. Therefore, the energy fed into the system is significantly higher, resulting in more heat being generated during cutting. The increases in chip thickness due to increasing the feed rates mean that the area of contact between the chip and tool increases and that there is more area to add friction resistance, particularly along the rake face, which adds to the increasing amounts of heat generated due to friction in the secondary shear zone (tool-chip interface)^57^. Although each edge of the tool is in contact with a point on the workpiece for a shorter duration at higher feeds, the mechanical load is higher for each revolution. This means there is greater heating localized to the edge of the tool. At higher feeds, the chips are thicker, less curled, and also less effective in moving heat away^58^. Consequently, thermal build-up is more likely to occur near the tip of the tool.

##### *Nanofluid (CuO and Al*_*2*_*O*_*3*_*) concentration*

Hybrid nanofluids are innovative cutting fluids, and this research employs two types of nanofluids, including copper oxide (CuO) and aluminum oxide (Al_2_O_3_) in a base fluid, typically in a water-glycerol mixture. These fluids are used increasingly in cutting processes, where enhanced thermal conductivity and lubrication at the tool-chip and tool-workpiece interfaces can have an advantageous effect^59^. The thermal and rheological properties of CuO and Al_2_O_3_ was beneficial in these hybrid nanofluuids since CuO provides high thermal conductivity to obtain heat from the cutting zone in a favorable manner, and Al_2_O_3_ provides excellent anti-friction properties that reduces resistance with the tool to chip. As such, hybrid nanofluids appear to serve as an excellent medium for cutting fluid in difficult-to-cut materials such as AISI 4340 steel, and provide extreme benefit during high-speed CNC turning, where appropriate thermal management is critical. As illustrated in Fig. 7c, e, and f, the increase in concentration of hybrid nanofluids (0.25–0.45%) is effective in reducing the tool tip temperature. The increased concentration increases the thermal conductivity of the fluid, thus allowing it to transport heat away from the cutting zone. As the number of nanoparticles increases, it also enhances the tribological performance of the lubricant, decreasing friction at the tool-chip interface and heat generated. At higher concentrations (up to 0.45%), the nanofluid will also produce a stable and uniform lubricating film on the tool surface, minimizing direct metal-to-metal contact, as well as reducing adhesion-related heating. But when the hybrid nanofluid concentration is increased from 0.45 to 0.5%, a change occurs, and instead of decreasing, the tool tip temperature begins to increase. This seemingly contradictory situation is obtained because undesirable effects become dominant with increased nanoparticle concentration. One main undesirable effect is the agglomeration of nanoparticles by Van der Waals forces. Agglomerates decrease the effective heat transfer surface area, obstruct flow in spray nozzles, and degrade cooling capability^60^. In addition, increasing particle size increases the viscosity of the nanofluid, which reduces its flowability and leads to poorer spray atomization^61^. The outcome is decreased fluid penetration and poor heat-dissipating qualities. Furthermore, excess nanoparticles accumulate on the tool surface and act as a thermal barrier on the tool instead of cooling the cutting edge. By limiting heat transfer away from the tool, the presence of excess nanoparticles adds to localized thermal buildup. High percentages of nanoparticles can also clog the delivery system, meaning input force will cause non-uniform distribution of fluid and sporadic cooling, further helping increase temperature. So, while moderate concentrations of hybrid nanofluids improved cooling and lubrication, excess concentrations may hinder thermal management.

##### Depth of cut

The increased depth of cut in the CNC turning of AISI 4340 steel from 0.4 mm to 0.8 mm greatly affects the temperature of the tool tip, as shown in Fig. 7b, d and f. This is due to the thermomechanical nature of the machining process and the material’s response to deformation. With increased DoC, the MRR increases, which means there is more plastic deformation per desired unit of time^62^. More deformation dissipates more energy, which then generates more heat. The increase in DoC also increases the chip cross-sectional area, increasing the normal and shear cutting loads. Greater force increases friction and plastic deformation, particularly in the primary shear zone and in the tool-chip interface region adjacent to the tool nose^63^. In addition, a larger depth of cut increases the contact area that exists between the tool and the workpiece and also the chip, leading to an increase in frictional heating (especially near the tool nose)^64^. The rate of heat being generated far exceeds that of heat being dissipated, and the tool temperature can reach high levels. Additionally, at maximum depth of cut, there is more of the tool nose and rake face in contact with the workpiece, so the thermal load is distributed over a larger area. Thus, the depth of cut and several other factors (cutting speed, feed rate, and nanofluids, CuO and Al_2_O_3_ concentration) must be optimized to obtain the best performance and machining results.

#### Surface roughness

##### Cutting speed

**Fig. 8:**
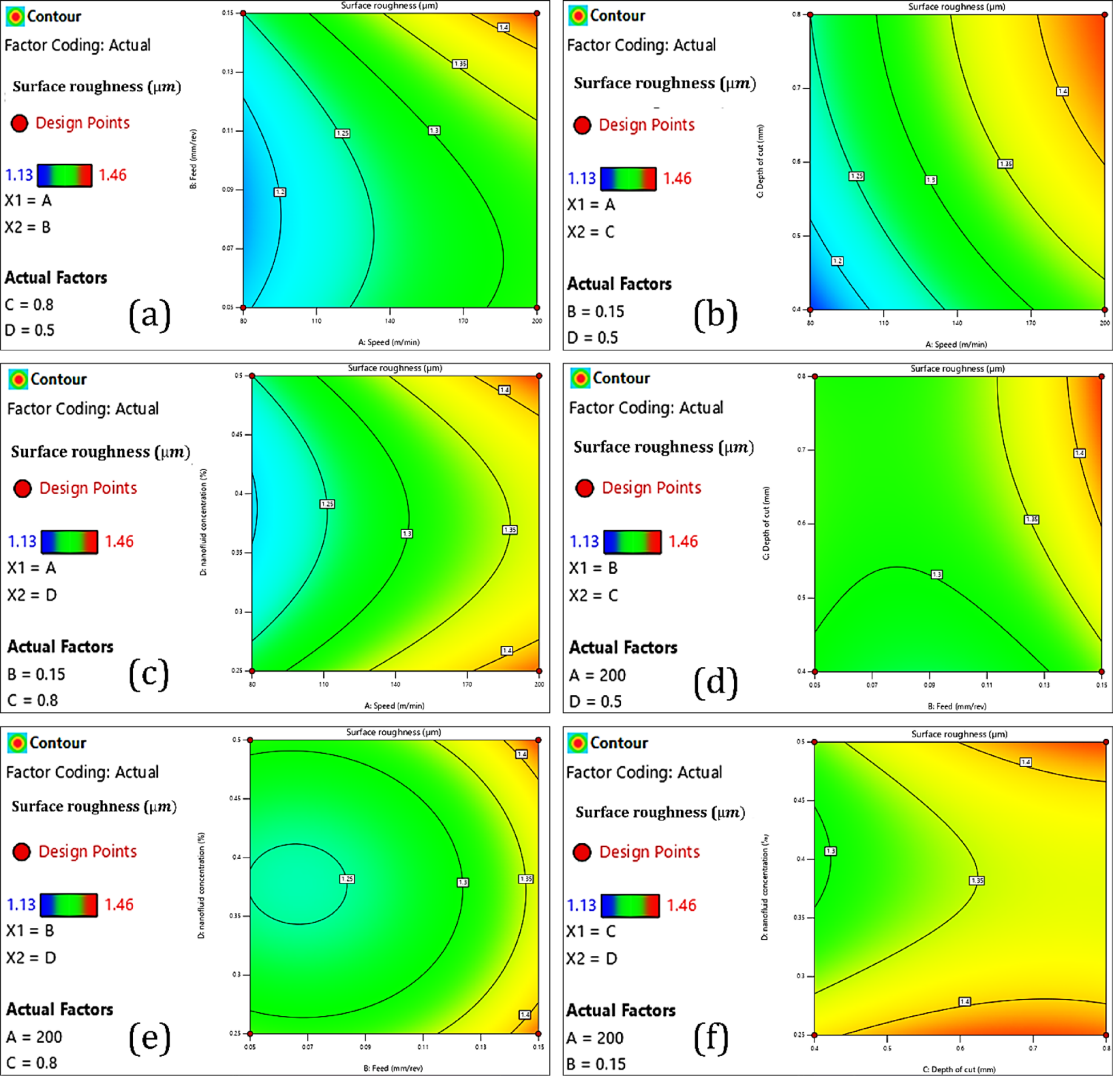
Interaction analysis of input parameters with surface roughness.

Cutting speed is an important process parameter in CNC turning that significantly affects the surface quality of the machined component. Surface integrity of AISI 4340 is an important consideration for most machining applications, both because it is an aerospace, automotive, and structural-specified grade of high-strength low-alloy steel, and due to the direct influence that surface integrity has on fatigue performance, accuracy in dimensions, and overall part operation. As seen in Fig. 8a, b and c, increasing cutting speed from 80 m/min to 200 m/min can result in a significant increase in surface roughness. Additionally, Fig. 10b–b3 displays the interaction effects of the input parameters on surface roughness. At lower cutting speeds (i.e., 80 m/min), the cutting zone temperature does not reach an extreme level, which can therefore stabilize chip formation and lead to controllable plastic deformation in the workpiece material. However, increasing cutting speed will significantly increase the temperature at the tool–workpiece interface. This will increase the thermal load at the cutting edge of the tool and, indirectly, could cause some softening of the workpiece surface, build-up edge formation on the cutting tool, and wear flat of the cutting-edge geometry^51^. These effects compromise the cutting action and cause a rougher surface finish. In addition, increasing cutting speed noticeably increases tool wear rates, particularly flank and crater wear, due to greater friction and thermal stress^65^. A cutting tool that has lost its sharpness for cutting will lead to a series of micro-vibrations and chatter throughout the entire machining process, resulting in a combined increase in surface roughness^66^. Furthermore, higher cutting speeds tend to cause dynamic instabilities, including tool deflection and higher mechanical vibrations^67^. These instabilities, which exist as moments of convergence, lead to the creation of waviness, chatter marks, or other surface deformations, further impacting surface quality. AISI 4340 also exhibits sensitivity to strain rates, which means it will produce a differing mechanical response based on the rate of deformation of the material^23^. When the cutting speed is higher, strain rates increase, which changes the morphology of the chip and produces less controlled shearing of the material^68^. As a result, the inconsistent mechanism of material removal causes unwanted surficial features with an associated roughness value. Although a higher cutting speed will allow greater productive cutting of material, this is not without some sacrifice in surface quality of the AISI 4340 material. The importance of a balanced consideration in selecting parameters when machining AISI 4340 material becomes more apparent. One possibility is the incorporation of advanced cooling, including but not limited to nanofluids as coolants that promote surface finish.

##### Feed rate

The feed rate significantly influences CNC turning and will influence the surface roughness of the machined workpiece. In most cases, increasing the feed rate diminishes the surface finish due to more apparent feed marks and a rougher material removal profile for the machining process. However, the results Fig. 8a, d and e demonstrate a more complex relationship. When the feed rate is increased from 0.05 mm/rev to 0.07 mm/rev, Ra increases. This result fits expected machining theory, as increased feed rates generally yield larger distances of separation between tool passes, creating a more textured and sporadic surface^57^. Interestingly, when the feed rate is increased from 0.07 mm/rev to 0.10 mm/rev, the surface roughness is slightly decreased. This identified anomaly may be the result of greater chip evacuation and process encasement at this feed rate^69^. It is possible that the cutting dynamics became more moderate in this region, potentially reducing vibrations and yielding a smoother material removal. However, as the feed rate increases from 0.10 to 0.15 mm/rev, there is a sharp increase in surface roughness, with the largest Ra value at 0.15 mm/rev. At that feed rate, the volume of material removed per revolution causes cutting forces to pile up and produce deflections and instabilities. All these non-ideal conditions promote negative processes such as ploughing, tearing, or chatter that can ultimately degrade the surface finish. The observation of this non-linear trend demonstrates that surface roughness is generally on an upward trend with the feed rate, but certain ranges exhibit transitional behavior due to dynamic changes in the chip formation process and their respective interaction with cutting forces^6^. This emphasizes the level of importance when defining and selecting feed rate parameters to create the appropriate balance between machining efficiency and surface finish, especially when cutting AISI 4340 high-strength steels.

##### Nanofluid (CuO and Al_2_O_3_) concentration

The applicability of nanofluids as cutting fluids in CNC turning has been shown to offer great opportunities for improved surface finish by enhancing lubrication and cooling at the tool–workpiece interface. In this study, hybrid nanofluids with copper oxide (CuO) and aluminum oxide (Al_2_O_3_) nanoparticles were used to examine the effect of concentration (from 0.25 to 0.5% by volume) on the surface roughness of AISI 4340 steel. As seen from Fig. 8c, e and f, it was found that increasing hybrid nanofluid concentration from 0.25 to 0.45% (by volume) significantly decreased surface roughness. It is postulated that this effect is due to improvements to thermophysical properties of the nanofluids, particularly improvements to thermal conductivity and lubricity, resulting in reductions in both cutting temperature and friction at the tool–workpiece interface^70^. The deposition of CuO and Al_2_O_3_ nanoparticles is likely to produce a protective tribofilm on the cutting tool surface, resulting in decreases in adhesive wear and a more uniform chip generation^59^. Collectively, these effects can result in decreases in machine surface roughness. However, when the concentration of the nanofluid was increased further than 0.45%, the surface roughness started to increase. Large amounts of nanoparticle loading may result in increased fluid viscosity and prevent sufficient fluid flow and penetration of the cutting edge with coolant^71^. Additionally, excessive concentration may cause aggravated nanoparticle agglomeration to occur, leaving the dispersion in an unstable state, thus clogging the inputs that supply coolant^72^. When the concentration of nanofluid is not at an optimal level, it will lower the cooling and lubrication performance, thereby increasing friction, worsening the chip’s formation, and creating a rough surface finish. The potential for hybrid nanofluids to enhance surface quality (at an optimal level) will necessitate careful consideration of the concentration in actual cases of precision machining.

##### Depth of cut

Depth of cut is a primary machining parameter that is directly related to the cutting forces, tool–workpiece interaction, and, as a result, surface finish of the machined part. In this study, the influence of increasing depth of cut on surface roughness was analyzed during the CNC turning of AISI 4340 steel. As shown in Fig. 8b, d and f, the results show that increasing the depth of cut from 0.4 mm to 0.8 mm directly influences surface roughness. This is primarily due to the increased cutting forces and heat produced at higher material removal rates^73^.

When increasing the depth of cut, the cutting tool engages a larger volume of material, which not only increases the mechanical load on the cutting tool but also increases friction at the tool–chip and tool–workpiece interfaces. The increased mechanical and thermal load can lead to numerous negative outcomes for the surface finish. An increase in depth of cut causes tool deflection and vibration when slender geometries are utilized or when working with a high-strength material like AISI 4340^74^. This dynamic instability shows chatter marks or waviness, causing surface roughness. Additionally, at larger depths of cut, cutting temperature is higher, aiding in tool wear, primarily flank and crater wear, which deteriorates the cutting edge and ability to produce a smooth surface^75^. The deeper cuts will often generate larger and more discontinuous chips, and this may ultimately lead to chip clogging or an erratic flow of the chips, which also degrades surface quality. The mechanical loading during machining leads to irregularities in the uniform removal of the material and an increase in the roughness of the workpiece surface. Therefore, while depth of cut can improve material removal efficiency and reduce machining time, it comes at the expense of surface integrity. An optimal depth of cut should be selected regarding productivity versus surface quality, especially in hard-to-cut alloys like AISI 4340^67^.

#### Material removal rate (MRR)

##### Cutting speed

**Fig. 9:**
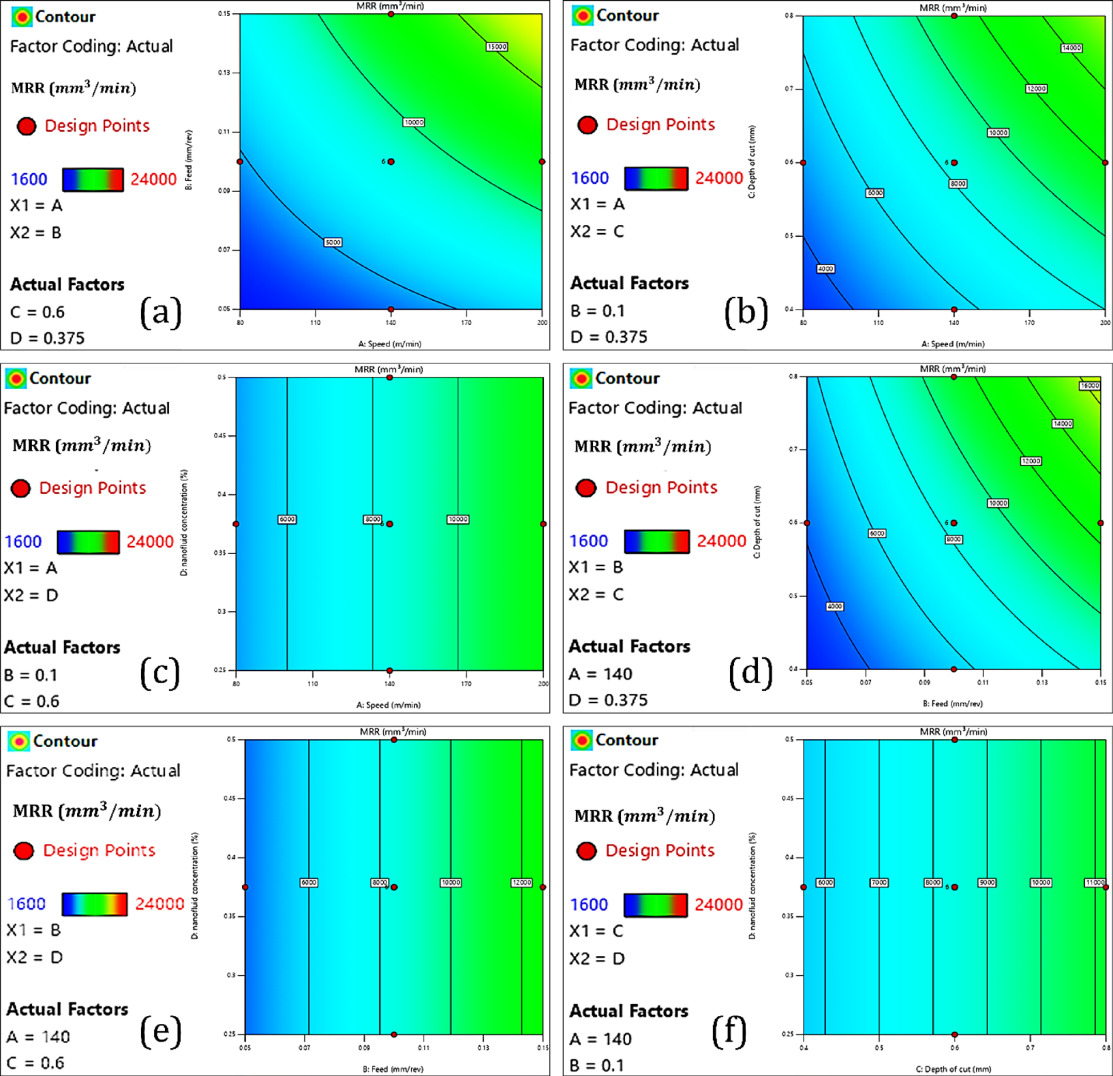
Interaction analysis of input parameters with MRR.

**Fig. 10:**
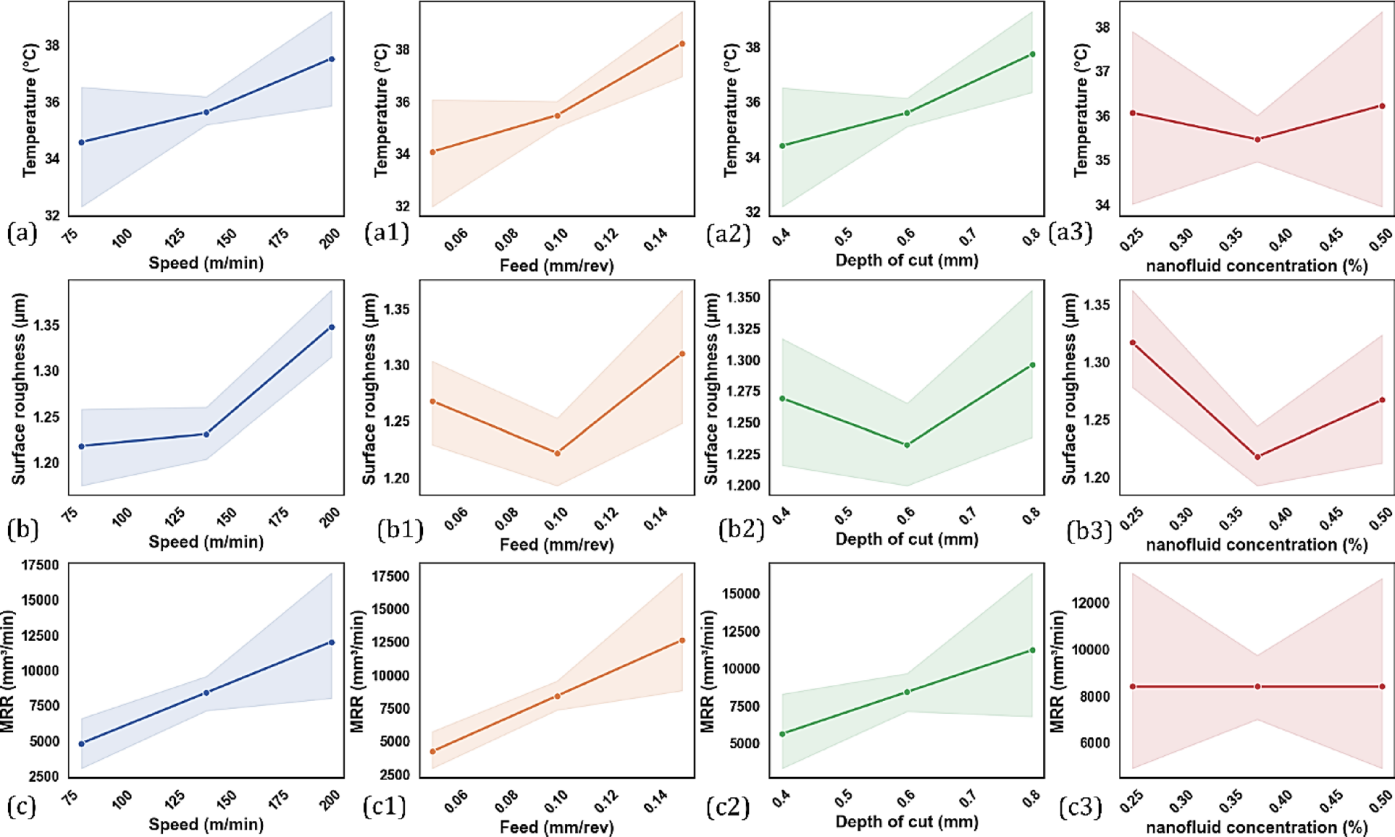
Influence of each input parameter on each response.

Cutting speed is a vital parameter in CNC turning and has a profound effect on machining performance, especially MRR. MRR is the amount of material removed over time, and an important measurement of productivity. As illustrated in Fig. 9a, b and c, increasing the cutting speed from 80 to 200 m/min caused a significant increase in MRR in the machining of AISI 4340 steel. Additionally, the interaction effects of the input factors on MRR are depicted in Fig. 10c–c3. The fact that MRR is a product of cutting speed, feed rate, and depth of cut is the primary cause for this increase in value. At the higher speed, the tool is engaged with the workpiece for a shorter time and contacts more workpiece material, resulting in acceleration of the MRR. This means less machining time and improved throughput, which are both very important in production or time-sensitive applications. The findings for this research confirm this pattern, as the change in MRR is nearly linear or progressive for each increase in cutting speed^76^. However, performance enhancements should be considered against the possible consequences. Fast cutting speeds create heat, and, as speed increases, so does heat generation, which can increase tool wear and surface integrity issues (if uncontrolled). Therefore, a tool is more likely to benefit from cooling approaches such as nanofluid-based lubrication if the intention is to allow high-speed machining to achieve maximum speed without negatively impacting tool life or part integrity.

##### Feed rate

Feed rate is an important process variable in CNC turning that impacts productivity and machining efficiency. The increase in MRR during the machining of AISI 4340 steel from a feed rate of 0.05 mm/rev to 0.15 mm/rev, as illustrated in Fig. 9a, d and e, is significant. An increase in feed rate means the cutting tool will remove a large volume of material for each revolution, resulting in higher MRR^77^. This is favorable as more time is saved from the process, which provides competitive advantages in a high-productivity industrial context. Nonetheless, while increasing the feed rate provides advantages to productivity, increasing the feed rate may also provide disadvantages to those benefits. Most significantly, higher feed rates will increase cutting forces and temperature at the tool–workpiece interface, which may accelerate wear on the tool and degrade surface finish^78^. These concerns are exacerbated when machining high-strength materials like AISI 4340, as the mechanical and thermal load presented on the cutting tool may be excessive. Accordingly, due consideration must be given to maximizing the MRR alongside adequate levels of surface integrity. Thus, while it may produce an advantage to efficiency, effective speed management alongside the combination of other process parameters must ensure optimal machining performance while also controlling component quality.

##### Nanofluid (CuO and Al_2_O_3_) concentration

The material removal rate is inherently determined by the product of the cutting speed, feed rate, and depth of cut. These machining parameters characterize the rate of material removed from the workpiece in some specified period. As nanofluid concentration cannot be part of this mathematical relationship, it does not affect MRR. As indicated in the obtained results shown in Fig. 9c, e and f, the increase in the concentration of the hybrid nanofluid (CuO and Al_2_O_3_) from 0.25 to 0.5% did not yield any significant change to MRR. Regardless of the concentration of the nanofluid, the MRR values were largely consistent. It makes reasonable that the material removal rate would stay constant independent of the coolant or lubricating medium, as the cutting speed, feed rate, and depth of cut were all the same throughout the research experiments. Nanofluids are thermally and tribologically significant in CNC turning but do not provide a volumetric contribution. Nanofluids have a fundamental role in improving cooling performance, lowering friction, and enabling tool–workpiece interactivity, which can facilitate more effective tooling life, improved surface finish, and better control of dimensional accuracy^71^. However, these characteristics do not intrinsically enhance MRR unless they allow for additional, more aggressive cutting parameters to be safely employed.

Nevertheless, this does not mean that hybrid nanofluids have no relationship to the material removal rate. Although hybrid nanofluids may not directly affect MRR, they can indirectly have a positive impact on material removal effectiveness because they can improve the thermal and tribological properties in the cutting zone. Improved cooling and lubrication can help to lower cutting forces, minimize tool wear, and increase working stability, which helps material removal occur more efficiently and consistently, especially when cutting parameters are further optimized^79^.

##### Depth of cut

The depth of cut is a crucial factor because it directly influences the MRR of CNC turning. The machining equation indicates that the material removal rate is a function of cutting speed, feed rate, and depth of cut. Therefore, if the other two parameters remained constant, increasing the depth of cut results in a proportional increase in MRR. As illustrated in Fig. 9b, d and f, an increase in the depth of cut from 0.4 to 0.8 mm increases MRR. The findings were consistent with theoretical expectations, as cutting at a greater depth allowed the cutting tool to engage a larger cross-section of the workpiece material and remove more material during that pass^80^. A deeper depth of cut means higher mechanical and thermal loads on the tool and workpiece interface, and these higher stresses can lead to faster tool wear, generate more vibration, and severely impact surface quality when not controlled^81^. Thus, increasing the depth of cut means increasing MRR, but the depth of cut should be considered in conjunction with tool material, coolant strategy, and other cutting conditions to create virtual stability in machining. Consequently, the findings above are strongly indicative that increasing the depth of cut is a way to improve machining efficiency through higher MRR. However, maintaining a balance between productivity and tool performance will be critical to ensure successful high-performance machining of materials, such as AISI 4340.

### Observed and machine learning results and predictions

**Fig. 11:**
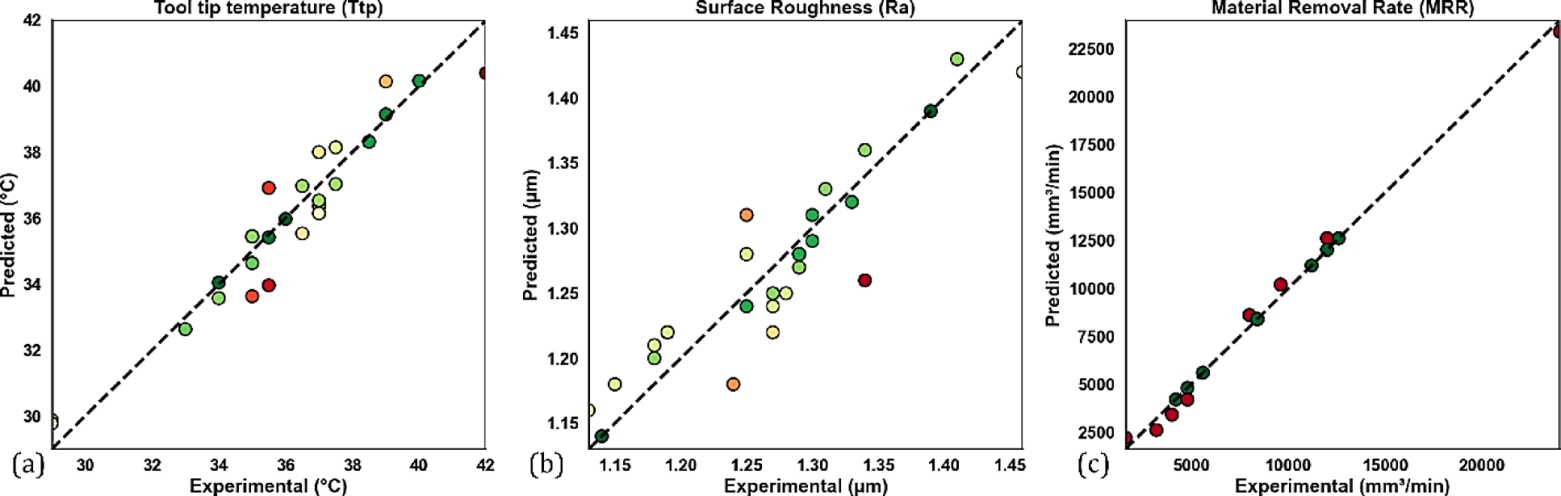
Comparison of experimental and RSM predicted results for (**a**) tool tip temperature, (**b**) surface roughness, and (**c**) MRR.

The comparison of experimental results with predictions made by the RSM is a very important step in assessing the validity of the RSM model. In this study, the experimental results for the tool tip temperature, surface roughness, and MRR with RSM predictions are compared as shown in Fig. 11a, b and c. The positions of the experimental data points and the RSM predicted values (the dotted points) in these figures further demonstrate how close the RSM predictions are to the experimental values (the reference line). The proximity between the experimental data values and RSM predictions indicates there is a negligible difference between the experimental data and predicted data, and that RSM provides a reliable model for real predictions.

**Fig. 12:**
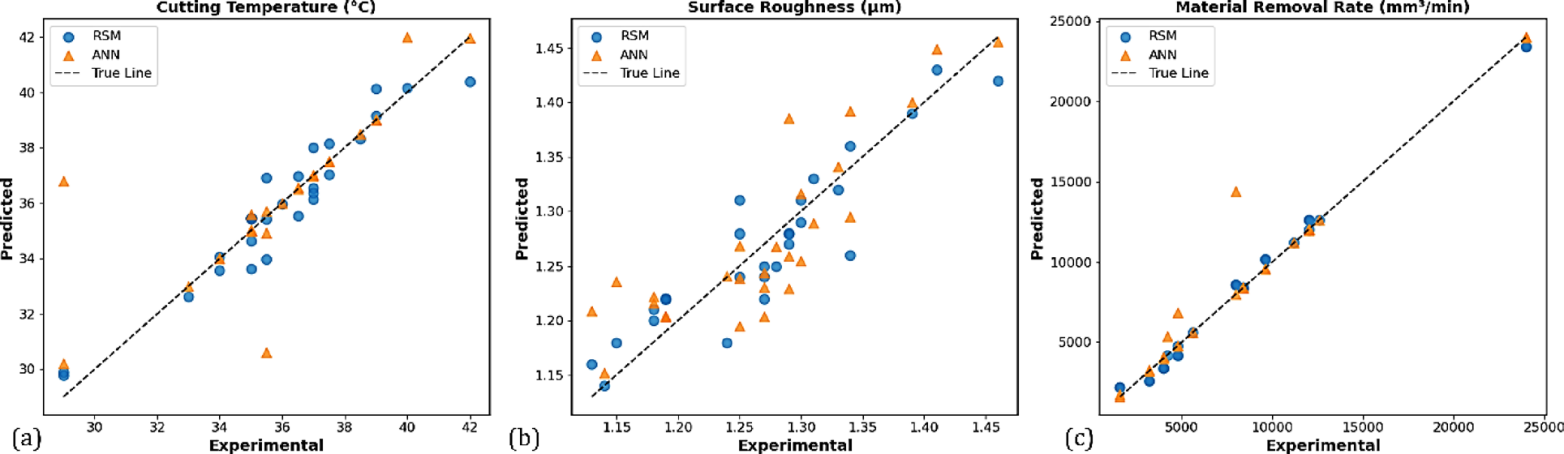
Comparison of RSM and ANN predictions with experimental results.

To confirm the predictive features of RSM, the RSM predictions were also compared to ANN predictions, as shown in Fig. 12. The ANN predictions show an identical trend and similar position to RSM and indirectly further validate the reliability of the experimental data. The alignment of ANN and RSM predictions emphasizes that RSM is a useful and reliable model for predicting process response^82^.

**Fig. 13:**
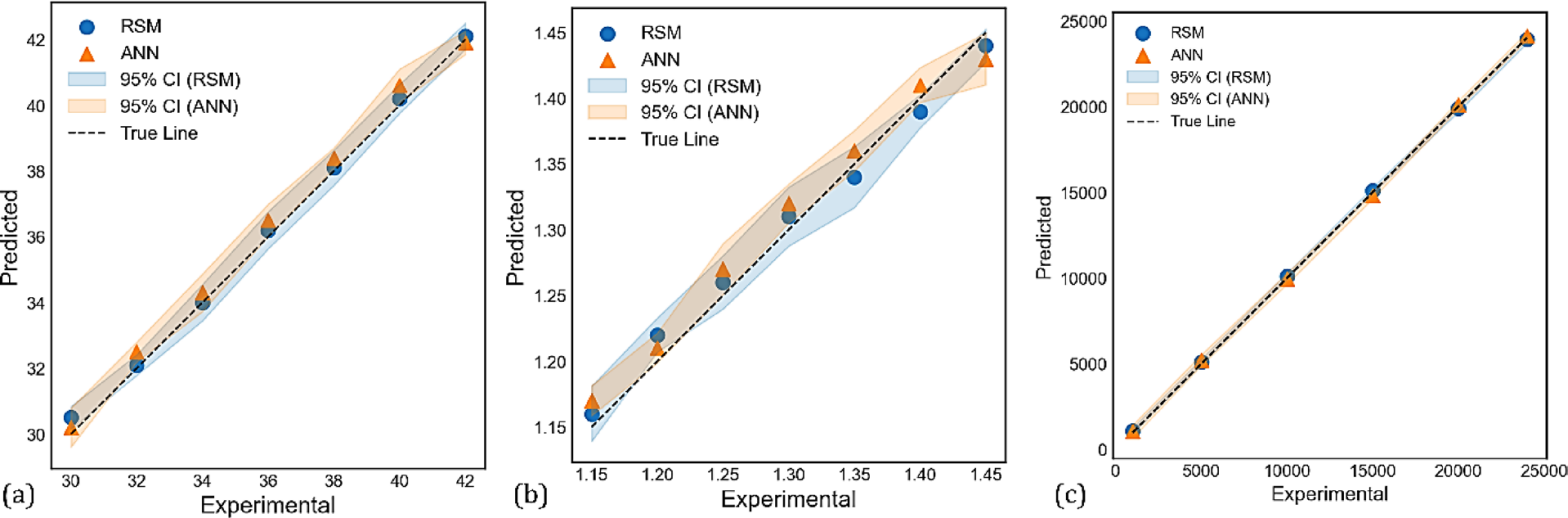
The 95% confidence intervals for (**a**) cutting temperature (°C), (**b**) surface roughness (μm), and (**c**) MRR (mm^3^/min) predicted by RSM and ANN models.

**Fig. 14:**
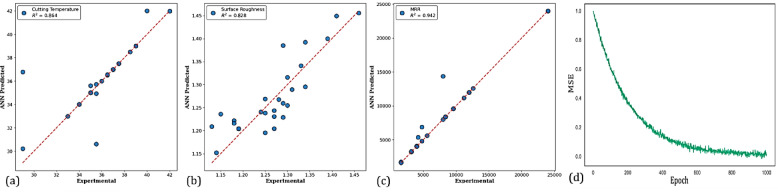
ANN predictions.

The accuracy of each result is discussed using the confidence intervals that are presented in Fig. 13, which provides details regarding the accuracy of the predictions obtained using an RSM and ANN model. The results for predicting cutting temperature demonstrate strong predictive performance, as evidenced by the data points closely aligning with the ideal true line in Fig. 13a. The confidence intervals for both methods are observed to overlap and encompass the true line, indicating that their predictions. However, confidence intervals associated with ANN appear to be a bit wider compared to those of RSM, pointing toward some uncertainty in the ANN predictions. Al-Ani^83^ notes that although ANN is more accurate, RSM generally gives narrower intervals and more stable predictions under certain experimental conditions, whereby the method of RSM would be preferred. Nevertheless, the reliability of this parameter is limited since both models can capture variations in cutting temperature. The analysis for surface roughness is presented in Fig. 13b. Comparatively, the predictions show some deviation from the true line than from the cutting temperature. While the RSM model’s predictions are more consistent with experimental observations, the ANN model occasionally overestimates surface roughness, particularly in the mid-range. There is some uncertainty because the ANN’s confidence interval is larger than the RSM’s. This demonstrates that, under the test conditions employed, RSM is more reliable and accurate in predicting surface roughness. Figure 13c presents the experimental versus predicted MRR. Both models performed exceptionally well in this plot, as predicted points lay almost entirely on the true line, indicating high accuracy and low uncertainty. This means that both modeling strategies have been able to predict the MRR very well, making it a valuable metric. Therefore, both RSM and ANN determine similar reliable predictions through their confidence interval limits, usually tighter for RSM, with generally more consistent accuracy than ANN, especially with surface roughness. The close agreement between predictions and experimental results across all parameters demonstrates the robustness of both techniques. The predictive performance of the ANN model is also assessed using the coefficient of determination (R^2^), as shown in Fig. 14a–c. The R^2^ values for tool tip temperature (0.864), surface roughness (0.828), and MRR (0.942) indicate that there is a strong correlation between the predictions of the ANN model and the experimental results. The Mean Squared Error (MSE) was also observed to decrease with increasing epochs; some of these values were less than 1 (Fig. 14(d)).

The decline in loss shows the ANN model gets more accurate with greater training iterations. MSE values nearing 1 demonstrate that the ANN model was able to sufficiently model the complex relationships among the process parameters and outputs, indicating that it was accurate and robust. Furthermore, the machine learning results shown in Fig. 14a–d demonstrate high model effectiveness. This is confirmed by R^2^ values approaching 1, along with minimal MAE and MSE, which collectively indicate strong predictive accuracy.

**Fig. 15:**
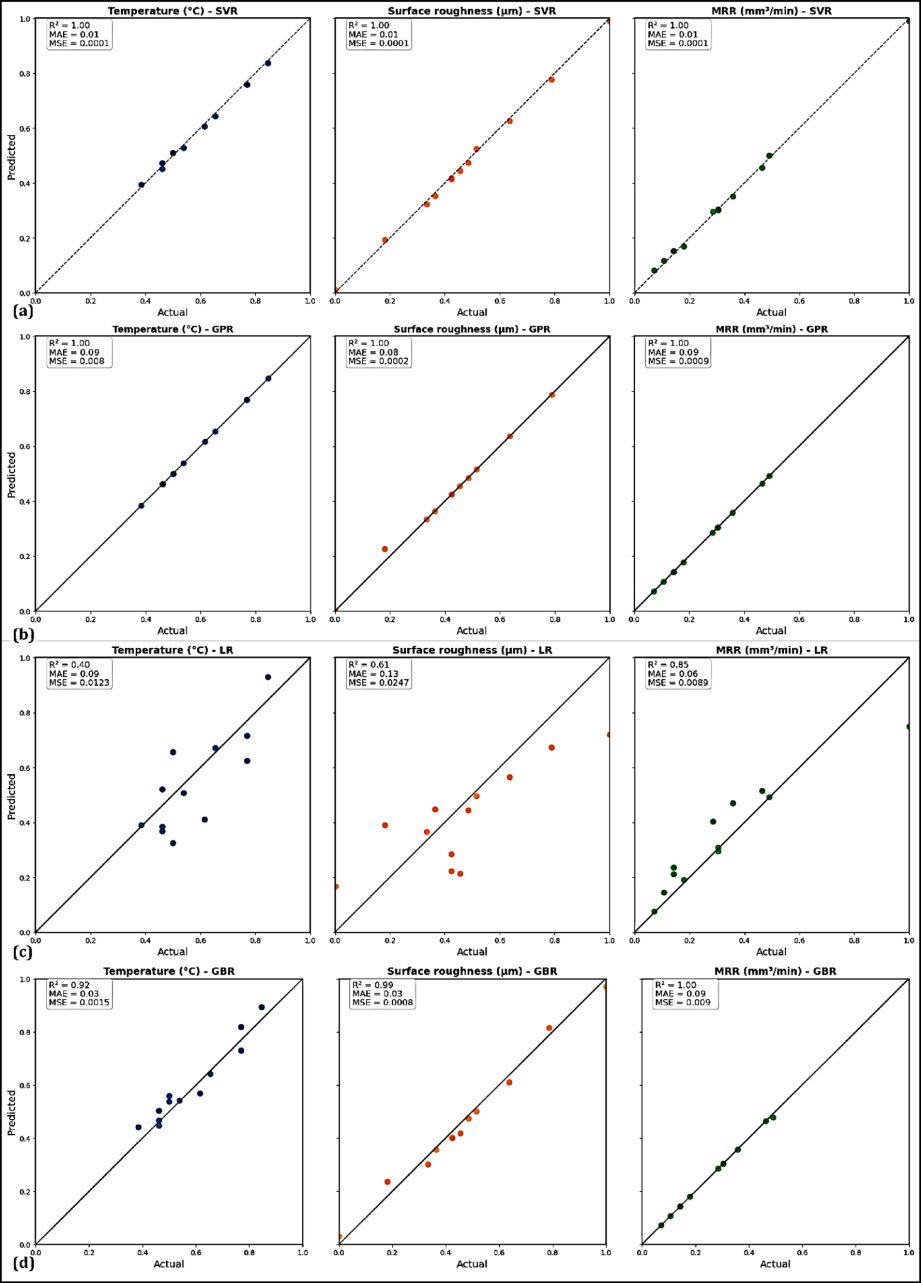
Machine learning model results: (**a**) SVR, (**b**) GPR, (**c**) LR, (**d**) GBR.

Furthermore, a clear indication of the predictive capability of the four ML models applied in this study is displayed in the results presented in Fig. 15. In Fig. 15a, SVR is claimed to perform exceptionally well, as the predicted values of temperature, surface roughness, and MRR show an almost perfect alignment with experimental data. They use statistical indicators, namely the R^2^, which is consistently equal to 1.00, and error values close to zero, to prove that SVR captured the nonlinear interactions ruling in the machining process without almost any deviation^84^. Figure 15b also shows that GPR predicted with nearly identical precision, R^2^ is 1.00, and with very low error levels across all responses. Although its MAE is slightly higher than for SVR, the model’s ability to handle nonlinearities is strong, since both errors are negligible compared to the size and scale of the data. However, Fig. 15c demonstrates the limitation of LR, that is, the predicted temperature and surface roughness are far from the reference line. Temperature and surface roughness have weak prediction capabilities with R^2^ values of 0.40 and 0.61, respectively, despite the comparatively superior R^2^ of 0.85 for MRR, which is still well below the level of non-linear models. The key issue is that the linear assumptions of the model greatly underestimate the complex interrelationship of parameters within these processes. Figure 15d depicts the performance of GBR, which has very good predictions for all three responses. The model’s predictions for temperature give rise to slight deviations, with R^2^ equal to 0.92, surface roughness being captured even better (R^2^ = 0.93) and MRR predicted to almost perfection (R^2^ = 1.00). This confirms that GBR can learn non-linear relationships when combined with ensemble learning, providing reliable and accurate outcomes. The findings further demonstrate that nonlinear regression models outperform linear methods in predicting machining response. The most accurate in this regard is SVR, which is closely followed by GPR. GBR, which performs at the same level, shows less variability in some responses. LR, on the other hand, consistently underperformed due to its inability to capture those nonlinear relationships, therefore rendering it unsuitable for accurate prediction. Thus, the combined findings demonstrate that the ML models used produced extremely precise predictions and closely reflected the physical enhancements observed during the machining of 4340 steels using hybrid nanofluids.

### Optimal results predicted by genetic algorithm (GA)

**Fig. 16:**
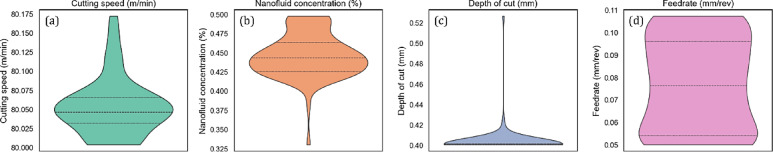
GA-predicted independent variables.

This section includes the maximum optimal machining conditions and the corresponding performance outcomes that were predicted by the GA used in this study. The result is presented using violin plots, which reflect the distribution of results and include summary statistics that allow one to see the various trends within the optimization. Concerning Fig. 16a–c, the violin plots represent the criteria that were optimized in terms of distribution of the input parameters, such as cutting speed, hybrid nanofluid concentration (CuO and Al_2_O_3_), depth of cut, and feed rate. The GA-based optimization determined the most optimum ranges that always remained narrow surfaces within the criteria built into the model. For cutting speed, the optimum converged around 80 m/min, which indicated this cutting speed would find a reasonable compromise for maximizing the material removal rate and heat generation/thermal properties of the workpiece while maintaining surface quality.

This matches the literature, which suggests that medium-to-high cutting speeds provide a compromise during machining of steels or alloys, keeping in mind both tool life and productivity^85^. For hybrid nanofluid concentration, it appears that the GA determined that an optimal concentration of around 0.4% would be adequate for both cooling and lubrication improvement. The ideal cooling and lubrication conditions were obtained to be close to 0.4%, a value substantiated by reviews which mention the normal optimal range for nanoparticles in nanofluids, usually between 0.25 and 0.6%^86^. Additionally, the tribological and thermal conductivity of hybrid nanofluids were always superior of the mono nanofluids.

**Fig. 17:**
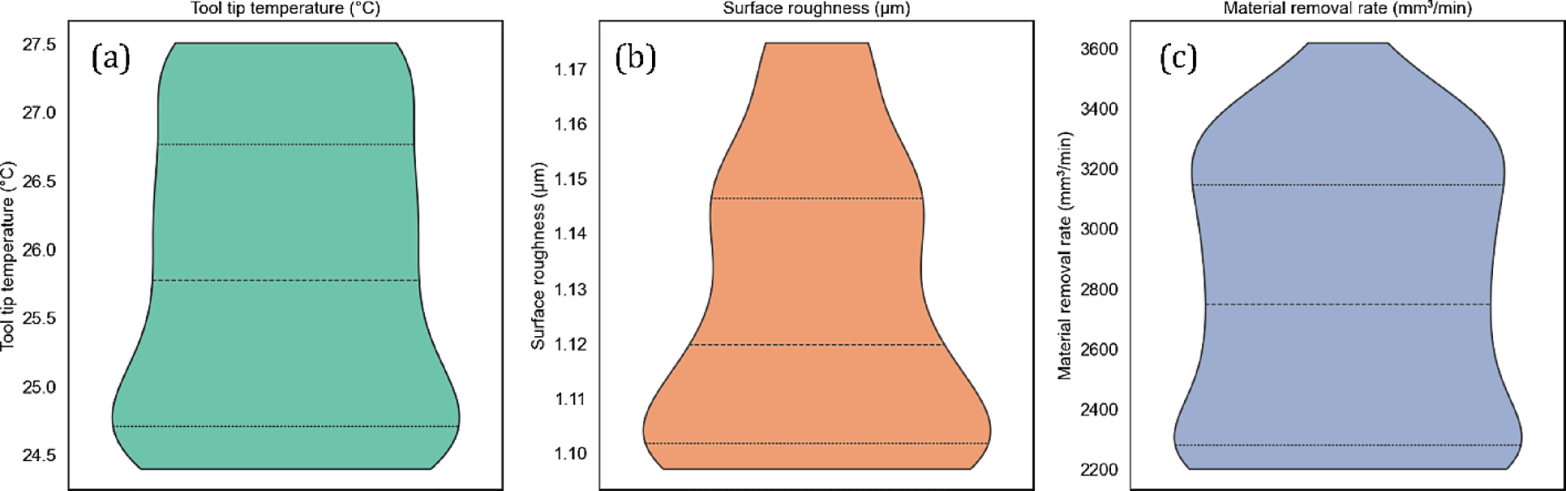
GA-predicted dependent variables.

**Fig. 18:**
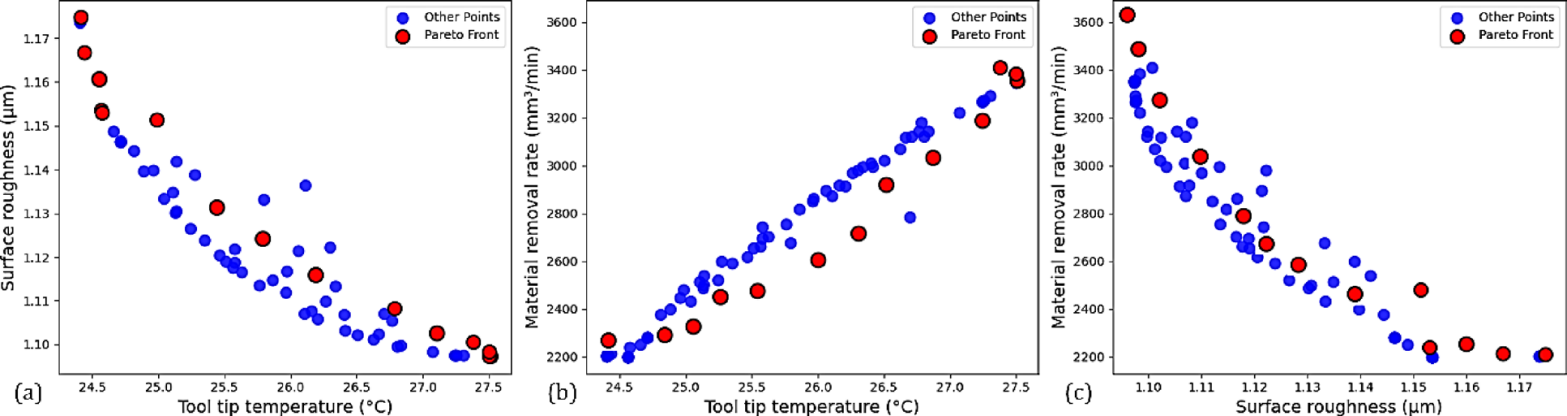
Pareto front.

The optimal cutting depth was found to be approximately 0.4 mm, where reasonable cutting forces and high MRR were balanced. This remarkable discovery was made in earlier research^85,87^, which emphasized the importance of depth of cut in assessing tool wear and MRR. Additionally, the feed rate of 0.07 mm/rev was optimal. This outcome was consistent with previous research that recommended low feed rates to achieve a more practicable productivity level with the minimum possible surface defects^88^. The resulting output parameters of tool tip temperature, surface roughness, and MRR are presented as violin plots in Fig. 17a–c. The tool tip temperature ranged between 24.4 and 27.5 °C, indicating that heat was managed efficiently, likely due to the hybrid nanofluid’s thermal conductivity. Surface roughness was consistently low, ranging from 1.09 and 1.17 µm, showing that the parameters chosen generate good quality surface finishes. The MRR ranged between 2201.5 and 3349.45 mm^3^/min, indicating adequate removal rates of material within a good operational range. The Pareto front plots shown in Fig. 18a–c illustrate the tradeoff between competing objectives by providing a visual aid to understand the behavior of multi-objective optimization problems, showing the non-dominated solutions within GA. Figure 18a presents the Pareto front that compares the surface roughness (y-axis) over tool tip temperature (x-axis). The curve slopes downward toward the bottom-right from its starting point in the upper-left. Because heat is insufficient to flow smoothly through chips, this negative slope suggests that lowering the tool tip temperature may increase surface roughness.

Therefore, there needs to be a balance between doing thermal control and producing high-quality surfaces. Figure 18b presents the Pareto front that compares the MRR (y-axis) over the tool tip temperature (x-axis). In this case, the curve begins at the top right and extends to the bottom left corner of the plot. The trade-off suggests that higher MRR generates higher temperatures, most likely due to the increased cutting forces and frictional resistance; thus, considerations should focus on thermal management during high productivity circumstances. The relationship between MRR (y-axis) and Ra (x-axis) is summarized in Fig. 18c. The Pareto front starting from the upper left and going to the lower right direction is consistent with the shifting trade-off in machining between MRR and surface finish. This shows that MRR and surface finish typically represent a trade-off relationship. This trade-off is common in machining and poses a challenge for machine operators and designers as they attempt to maximize MRR while preserving surface integrity. These Pareto plots illustrate the conflicting effects of machining objectives, thereby demonstrating the utility of evolutionary multi-objective optimization approaches such as GA. The shape and direction of each Pareto front provide actionable information for decision makers who would like to identify process parameters that would be optimal for a given trade-off between productivity, surface quality, and thermal effects. Consequently, these findings support the notion that GA is a valid way of solving complicated optimization problems associated with multi-objective optimization problems in machining^85^. Additionally, the application of hybrid nanofluids with evolutionary optimization techniques like GA represents a valid option for undertaking improvements to the thermal, surface, and productivity performance of machining applications and contributing to the modern landscape of machining.

### Validation

To validate the results obtained, the predictions of the ANN model were compared with the experimental values of 10 runs chosen from 30 total runs. The error metrics for cutting temperature, surface roughness, and MRR were found from Table 6 to be 0.87%, 1.87%, and 1.68%, respectively.

**Table 6:** Validation.

Run	${V}_{c}$	$f$	${a}_{p}$	${C}_{nf}$	Experiment			ANN			Error		
					${T}_{tp}$	Ra	MRR	${T}_{tp}$	Ra	MRR	${T}_{tp}$	Ra	MRR
21	200	0.05	0.4	0.250	35.5	1.34	4000	35.7	1.39	4084.7	0.56	3.59	2.070
22	200	0.05	0.4	0.500	33.0	1.30	4000	32.9	1.31	4000.0	0.30	0.76	0.000
23	140	0.1	0.6	0.375	35.0	1.27	8400	34.9	1.26	8399.9	0.28	0.78	0.001
24	140	0.05	0.6	0.375	35.5	1.25	4200	34.9	1.19	4353.3	1.69	4.80	3.521
25	80	0.15	0.4	0.250	35.0	1.27	4800	35.5	1.23	5181.2	1.40	3.14	7.357
26	80	0.05	0.8	0.500	36.5	1.18	3200	36.5	1.21	3310.7	0.00	2.47	3.343
27	80	0.05	0.4	0.250	29.0	1.29	1600	30.2	1.31	1608.5	3.97	1.52	0.528
28	140	0.1	0.4	0.375	35.0	1.24	5600	35.0	1.24	5600.0	0.00	0.00	0.000
29	140	0.1	0.6	0.375	35.0	1.19	8400	34.9	1.20	8399.9	0.28	0.83	0.001
30	140	0.1	0.6	0.375	35.0	1.19	8400	34.9	1.20	8399.9	0.28	0.83	0.001
Average error (%)											0.87	1.87	1.68

**Fig. 19:**
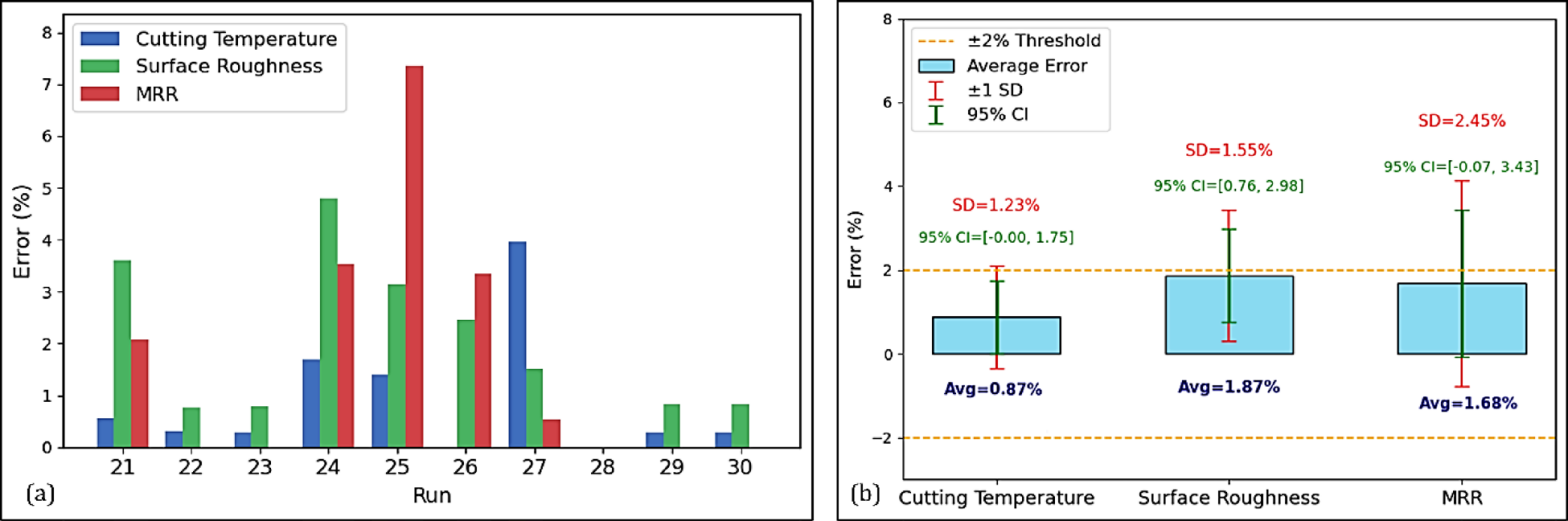
(**a**) Error of each response across chosen samples. (**b**) Error metrics with mean values, SD, and 95% CI.

This observation supports the validity of the ANN model and its experimental verification approach, as all average errors are significantly lower than the ± 2% threshold. Figure 19a shows the distribution of errors in these three responses from Runs 21–30. While some runs showed minimal error, others (such as Runs 25–26) showed significant variation from this threshold for both MRR and surface roughness, indicating the presence of variation among runs. A statistical analysis of the error metrics in terms of mean, standard deviations (SD), and 95% confidence intervals (CI) is shown in Fig. 19b. Variability in accuracy of predictions is shown by the SD values, which range from 1.23 to 2.45%, especially for MRR. The confidence of the ANN predictions is supported by the 95% CI, which, although somewhat wide, mostly remains within ± 2% threshold. It can thus be concluded that the results demonstrate that the proposed ANN model provides a very high predictive accuracy.

### Confirmation

**Fig. 20:**
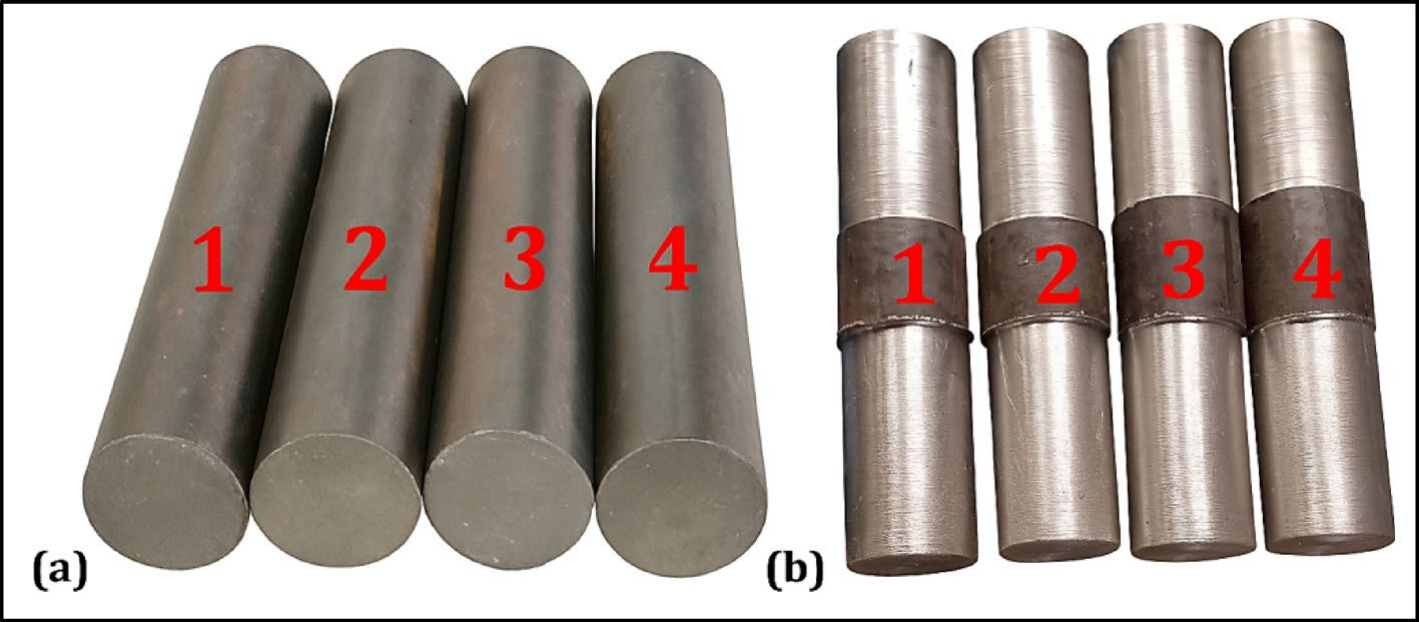
Samples for confirmatory analysis.

To confirm the optimized outcomes obtained in this research, a confirmation experiment was implemented with four confirmatory samples, as represented in Fig. 20a. The optimum input parameter values for the samples’ machining were 80 m/min for the cutting speed, 0.4% for the concentration of the nanofluid, 0.4 mm for the depth of cut, and 0.07 mm/rev for the feed rate. Figure 20b indicates the machined samples. The results of the confirmation experiments demonstrated in Table 7 demonstrate that most experimental values fall within the prediction ranges of the GA for tool tip temperature (24.4–27.5 °C), surface roughness (1.09–1.17 µm), and MRR (2201.5–3349.45 mm^3^/min).

**Table 7:** Confirmation.

Optimized inputs					Samples										
${V}_{c}$	${C}_{nf}$	${a}_{p}$	$f$	Test	Sample-1		Sample-2		Sample-3		Sample-4		Average		MRR
					${T}_{tp}$	Ra	${T}_{tp}$	Ra	${T}_{tp}$	Ra	${T}_{tp}$	Ra	${T}_{tp}$	Ra	
80	0.4	0.4	0.07	Exp	25.5	1.08	26.4	1.12	27.3	1.21	25.2	1.10	26.1	1.12	2240

The average values for each response are also closely aligned with the GA-predicted outcomes. These results justify the accuracy of the optimization method used in this study. The relationship between the predicted and experimental results confirms the validity and real-world usage of the optimized parameter settings.

## Conclusion

This study investigated the combined effects of machining parameters and concentrations of hybrid nanofluid (CuO & Al_2_O_3_) on tool tip temperature, surface roughness, and MRR during CNC turning of AISI 4340 steel. Based on the results, it was shown that the tool tip temperature is significantly increased by increasing the cutting speed, feed rate, and the depth of cut due to the increase in heat generated. Furthermore, the effect of the hybrid nanofluids was shown to greatly decrease the temperature effect at an optimum concentration. The tool tip temperature and the surface roughness were significantly reduced in the machining of AISI 4340 when the hybrid concentration was between 0.25 and 0.45%. When the hybrid concentration was above 0.45%, the tool tip temperature and surface roughness levels were again increasing. This could plausibly be explained due to the increased viscosity of the hybrid fluid at higher concentrations, which does not facilitate proper cooling and lubrication. A new technique for preparing and using hybrid nanofluid as a coolant was developed, found to be very effective and beneficial when conducting CNC turning of AISI 4340 steel, with superior thermal and tribological performance was achieved. The ANN modeling presented a very good prediction performance, producing R^2^ values of 0.864, 0.828, and 0.942 for the tool tip temperature, surface roughness, and MRR, respectively. The optimization with the GA approach outlined optimum machining parameters of a cutting speed of 80 m/min, nanofluid concentration of 0.4%, a feed rate of 0.07 mm/rev, and a depth of cut of 0.4 mm. These machining parameters achieved a reasonable compromise on controlling thermal temperatures, surface finish, and machining performance. Moreover, this is the basis for intelligent, adjustable machining settings, where the performance of hybrid nanofluids can be predicted and fine-tuned in real time, optimizing the process in terms of efficiency and sustainability for manufacturing applications. Validation through experimental trials confirmed that the optimization and modeling methods were accurate, as prediction errors for all response variables remained within ± 2%. Verification experiments added additional support to validation results that the optimized parameters provide stable and consistently high-performance machining processes. Thus, this research originally validated the significant importance of machining parameters and coolant formulation in enhancing performance, but also presented a new hybrid nanofluid preparation method that provides a feasible, efficient, and eco-friendly approach for the enhancement of CNC turning processes for difficult-to-machine materials (AISI 4340 steel).

Future research should focus on detailed characterization of hybrid nanofluids, such as viscosity, thermal conductivity, stability, tribological aspects, and their influence on machining performance. Research should also explore diverse hybrid nanofluid formulations by varying nanoparticle types and concentrations to optimize thermal management, lubrication, and tool life in machining. With real-time monitoring integrated with AI-based control systems like ANN–GA, adaptive optimization of cutting parameters and nanofluid application can be achieved. Translating laboratory-scale results into industrial conditions will enable wider adoption, especially for difficult-to-machine materials.

## Data Availability

The datasets generated during and/or analyzed during the current study are available from the corresponding author on reasonable request.

## References

[1] Roy, S., Kumar, R., Das, R. K. & Sahoo, A. K. A comprehensive review on machinability aspects in hard turning of AISI 4340 steel. *IOP Conf. Ser. Mater. Sci. Eng.*10.1088/1757-899X/390/1/012009 (2018).

[2] Abdul Rahman, H., Jouini, N., Ghani, J. A. & Rasani, M. R. M. A review of high-speed turning of AISI 4340 steel with minimum quantity lubrication (MQL). *Coatings*10.3390/coatings14081063 (2024).

[3] Sivaraman, V. & Prakash, S. Recent developments in turning hardened steels - A review. *IOP Conf. Ser. Mater. Sci. Eng.*10.1088/1757-899X/197/1/012009 (2017).

[4] Ojolo, S. J., Adjaottor, A. A. & Olatunji, R. S. Experimental prediction and optimization of material removal rate during hard turning of austenitic 304L stainless steel. *J. Sci. Technol.* **36**, 34. 10.4314/just.v36i2.4 (2016).

[5] Bolar, G. & Joshi, S. N. Experimental investigation and optimization of wall deflection and material removal rate in milling thin-wall parts. *Manuf. Rev.*10.1051/mfreview/2021015 (2021).

[6] Ibrahim, M.R., Sreedharan, T., Aisyah Fadhlul Hadi, N., Mustapa, M.S., Emran Ismail, A., Hassan, M.F., Mubarak Tajul Arifin, A. The effect of cutting speed and feed rate on surface roughness and tool wear when machining D2 steel, In *Materials Science Forum 2017* (2017) 80–88. 10.4028/www.scientific.net/MSF.909.80.

[7] Liu, Z. Q., Wan, Y. & Liu, J. G. The impact of tool materials and cutting parameters on surface roughness in high-speed face-milling. *Key Eng. Mater.* **258–259**, 462–465. 10.4028/www.scientific.net/kem.259-260.462 (2004).

[8] Shihab, S. K. & Mubarak, E. M. Evaluation of surface roughness and material removal rate in end milling of complex shape, univers. *J Mech. Eng.* **4**, 69–73. 10.13189/ujme.2016.040303 (2016).

[9] Bui, G. T., Do, T. V., Nguyen, Q. M., Thi, M. H. P. & Vu, M. H. Multi-objective optimization for balancing surface roughness and material removal rate in milling hardened SKD11 alloy steel with Sio2 nanofluid MQL, Eureka. *Phys. Eng.* **2024**, 157–169. 10.21303/2461-4262.2024.003042 (2024).

[10] Somayaji, B. S., Bhat, R., Naik, N. & Rajendra, B. Optimization of turning parameters and cooling techniques for enhanced machining performance of EN8 steel using L9 orthogonal array. *Eng. Proc.*10.3390/engproc2023059243 (2023).

[11] Altaf, S. F., Parray, M. A., Khan, M. J., Wani, M. F. & Bhat, F. A. Machining with minimum quantity lubrication and nano-fluid MQL: A review. *Tribol Online* **19**, 209–217. 10.2474/trol.19.209 (2024).

[12] Shah, R., Shirvani, K. A., Przyborowski, A., Pai, N. & Mosleh, M. Role of nanofluid minimum quantity lubrication (NMQL) in machining application. *Lubricants*10.3390/lubricants10100266 (2022).

[13] Rifat, M., Rahman, M. H. & Das, D. A review on application of nanofluid MQL in machining. *AIP Conf. Proc.*10.1063/1.5018533 (2017).

[14] Sai, P. H. V. S. T., Raj, R. A. & Anand, V. G. K. Influence of nano-fluid MQL in machining processes: A review. *Technol. Innov. Eng. Res.* **2**, 151–162. 10.9734/bpi/tier/v2/6140f (2022).

[15] Tai, B., Stephenson, D., Furness, R. & Shih, A. Minimum quantity lubrication for sustainable machining. *Encycl. Sustain. Technol.*10.1016/B978-0-12-409548-9.10213-1 (2017).

[16] Rosli, N. & Zamiruddin, N. E. H. Application of minimum quantity lubrication for various machining processes – a mini review. *J. Mod. Manuf. Syst. Technol.* **4**, 40–47. 10.15282/jmmst.v4i2.5137 (2020).

[17] Patole, P.B., Kulkarni, V. V., Patil, A.S., Lokapure, R.B., Chakule, R.R. Sustainable Machining Using Minimum Quantity Lubrication with Nano Fluid. In *2023 IEEE Engineering Informatics* (2023). 10.1109/IEEECONF58110.2023.10520618.

[18] Gupta, A., Kumar, R., Kumar, H. & Garg, H. Sustainable machining using hybrid nanofluids under minimum quantity lubrication (MQL). *Lect. Notes Mech. Eng.*10.1007/978-981-13-6412-9_56 (2019).

[19] Lotfi, B., Namlu, R. H. & Kılıç, S. E. Machining performance and sustainability analysis of Al2O3-CuO hybrid nanofluid MQL application for milling of Ti-6Al-4V. *Mach. Sci. Technol.* **28**, 29–73. 10.1080/10910344.2023.2287655 (2024).

[20] Singh, A. P., Dwivedi, R. K. & Suhane, A. Assessment of performance impact of conventional lube oil enhanced with Al2O3 and CuO nanoparticles, individually and combined. *J. Dispers. Sci. Technol.*10.1080/01932691.2024.2448752 (2025).

[21] Karimbaev, R., Pyun, Y. S., Maleki, E., Unal, O. & Amanov, A. An improvement in fatigue behavior of AISI 4340 steel by shot peening and ultrasonic nanocrystal surface modification. *Mater. Sci. Eng. A*10.1016/j.msea.2020.139752 (2020).

[22] Das, A., Mukhopadhyay, A., Patel, S. K. & Biswal, B. B. Comparative assessment on machinability aspects of AISI 4340 alloy steel using uncoated carbide and coated cermet inserts during hard turning. *Arab. J. Sci. Eng.* **41**, 4531–4552. 10.1007/s13369-016-2160-0 (2016).

[23] Bin Rashid, W., Goel, S., Davim, J. P. & Joshi, S. N. Parametric design optimization of hard turning of AISI 4340 steel (69 HRC). *Int. J. Adv. Manuf. Technol.* **82**, 451–462. 10.1007/s00170-015-7337-2 (2016).

[24] Raof, N. A., Ghani, J. A. & Haron, C. H. C. Machining-induced grain refinement of AISI 4340 alloy steel under dry and cryogenic conditions. *J. Mater. Res. Technol.* **8**, 4347–4353. 10.1016/j.jmrt.2019.07.045 (2019).

[25] Fatima, A. et al. Comparative study of sol-gel and co-precipitation techniques for synthesizing Calotropis Procera-mediated bismuth ferrite for biomedical and environmental applications. *Res. Chem.*10.1016/j.rechem.2025.102149 (2025).

[26] Elsheikh, N. Y., Shams, M. S., Arais, A. A. & Battisha, I. K. Structural, morphological, and magnetic study of transition metals co-doped ZnFeO nanocomposites prepared by sol-gel method for spintronic applications. *Ceram. Int.*10.1016/j.ceramint.2025.01.448 (2025).

[27] Fakhimi, O., Najafi, A. & Khalaj, G. A facile rout to obtain Al2O3 nanopowder via recycling aluminum cans by sol-gel method. *Mater. Res. Express*10.1088/2053-1591/ab8653 (2020).

[28] Balogun, S. O., Yaro, S. A., Abdulwahab, M. & Kasim, A. Production and characterization of alumina nanoparticles from giro clay via acid leaching with sol gel method. *Futa J. Eng. Eng. Technol.* **15**, 1–10. 10.51459/futajeet.2021.15.1.261 (2021).

[29] Ziva, A. Z. et al. Recent progress on the production of aluminum oxide (Al2O3) nanoparticles: A review. *Mech. Eng. Soc. Ind.* **1**, 54–77. 10.31603/mesi.5493 (2021).

[30] Rocha, F. & Simões, S. Aluminum nanocomposites reinforced with Al2O3 nanoparticles: Synthesis, structure, and properties. *J. Compos. Sci.*10.3390/jcs8010033 (2024).

[31] Nagdalian, A. et al. Nano-priming of pea (*Pisum sativum* L.) seeds with CuO nanoparticles: Synthesis, stabilization, modeling, characterization, and comprehensive effect on germination and seedling parameters. *Food Chem.*10.1016/j.foodchem.2025.143569 (2025). 40037223 10.1016/j.foodchem.2025.143569

[32] Asangi, D. U. et al. Electrochemical investigation of Al2O3-CuO binary nanocomposite electrodes: Towards high energy storage supercapacitors. *J. Alloys Compd.*10.1016/j.jallcom.2025.180806 (2025).

[33] Suneetha, S., Subbarayudu, K. & Bala Anki Reddy, P. Hybrid nanofluids development and benefits: A comprehensive review. *J. Therm. Eng.* **8**, 445–455. 10.18186/thermal.1117455 (2022).

[34] Guo, Z. et al. Heat transfer characteristics and flow characteristics of Al2O3-CuO/Water hybrid nanofluids in hexagonal serpentine microchannel. *Heat Transf. Res.* **5**, 1–4. 10.1615/HeatTransRes.2025057473 (2025).

[35] Dilawar, M., Qayoum, A., Ahmad, M., Bhat, G. S. & Narayana, T. Performance assessment of POE/Al2O3–CuO hybrid nanolubricants in refrigeration system with predictive machine learning. *J. Therm. Anal. Calorim.*10.1007/s10973-025-14140-9 (2025).

[36] Efa, D. A., Dejene, N. D., Ifa, D. A., Nemomsa, S. K. & Gemechu, T. B. Improving computer numerical control (CNC) turning performance of AISI D2 steel with nanofluid composites and advanced machine learning techniques. *Int. J. Adv. Manuf. Technol.* **138**, 511–539. 10.1007/s00170-025-15536-5 (2025).

[37] Gemechu, L. D., Efa, D. A. & Abebe, R. Optimizing CNC turning of AISI D3 tool steel using Al₂O₃/graphene nanofluid and machine learning algorithms. *Heliyon* **10**, e40969. 10.1016/j.heliyon.2024.e40969 (2024). 39735623 10.1016/j.heliyon.2024.e40969 PMC11681877

[38] Mahamude, A. S. F. et al. A comprehensive review on efficiency enhancement of solar collectors using hybrid nanofluids. *Energies*10.3390/en15041391 (2022).

[39] Epandi, A. M., Tijani, A. S., Abdulrahman, S. T., Kubenthiran, J. & Muritala, I. K. Numerical simulation of thermophysical properties and heat transfer characteristics of Al2O3/CuO nanofluid with water/ethylene glycol as coolant in a flat tube of car radiator, Pertanika. *J. Sci. Technol.* **30**, 853–873. 10.47836/pjst.30.2.01 (2022).

[40] Ukueje, W. E., Abam, F. I. & Obi, A. A perspective review on thermal conductivity of hybrid nanofluids and their application in automobile radiator cooling. *J. Nanotechnol.*10.1155/2022/2187932 (2022).

[41] Hirudayanathan, H. P. et al. A review on influence of nanoparticle parameters on viscosity of nanofluids and machining performance in minimum quantity lubrication. *Proc. Inst. Mech Eng. Part E J. Process Mech. Eng.* **239**, 1005–1024. 10.1177/09544089231189668 (2025).

[42] Abellán-Nebot, J. V., Ameen, K. H., Khan, A. M. & Mondragón, R. Application of hybrid nanofluids in MQL assisted machining operations: Exploring synergies and establishing guidelines. *Int. J. Precis. Eng.– Manuf. Green Technol.* **12**, 657–689. 10.1007/s40684-024-00675-z (2025).

[43] Liu, Y. et al. Ultrastiff metamaterials generated through a multilayer strategy and topology optimization. *Nat. Commun.*10.1038/s41467-024-47089-8 (2024). 38582903 10.1038/s41467-024-47089-8 PMC10998847

[44] Zulkafli, R. W. & Wanatasanappan, V. V. Experimental investigation on the dispersion stability of Al2O3-CuO hybrid nanofluid using ultraviolet (UV)-visible spectroscopy and Zeta potential analyzer. *AIP Conf. Proc.*10.1063/5.0051492 (2021).

[45] Amin, A. R., Ali, A. & Ali, H. M. Application of nanofluids for machining processes: A comprehensive review. *Nanomaterials*10.3390/nano12234214 (2022). 36500836 10.3390/nano12234214 PMC9739788

[46] Nobrega, G. et al. Recent developments on the thermal properties, stability and applications of nanofluids in machining, solar energy and biomedicine. *Appl. Sci.*10.3390/app12031115 (2022).

[47] Kong, D., Chen, Y. & Li, N. Gaussian process regression for tool wear prediction. *Mech. Syst. Signal Process.* **104**, 556–574. 10.1016/j.ymssp.2017.11.021 (2018).

[48] Geetha, C. T., Dash, A. K., Kavya, B. & Amrita, M. Analysis of hybrid nanofluids in machining AISI 4340 using minimum quantity lubrication. *Mater. Today Proc.* **43**, 579–586. 10.1016/j.matpr.2020.12.083 (2020).

[49] Fedai, Y. Exploring the impact of the turning of AISI 4340 steel on tool wear, surface roughness, sound intensity, and power consumption under Dry, MQL, and Nano-MQL conditions. *Lubricants*10.3390/lubricants11100442 (2023).

[50] Patole, P. B. & Kulkarni, V. V. Experimental investigation and optimization of cutting parameters with multi response characteristics in MQL turning of AISI 4340 using nano fluid. *Cogent Eng.*10.1080/23311916.2017.1303956 (2017).

[51] Dos Santos, M. R. et al. Analyses of effects of cutting parameters on cutting edge temperature using inverse heat conduction technique. *Math. Probl. Eng.*10.1155/2014/871859 (2014).

[52] Akbar, F., Mativenga, P.T., Sheikh, M.A. An investigation of the tool-chip interface temperature and heat partition in high-speed machining of AISI/SAE 4140 steel with TiN-coated tool, In *Proceedings of the 35th International MATADOR Conference: Formerly The International Machine Tool Design and Research Conference* (2007) 215–218. 10.1007/978-1-84628-988-0_48.

[53] Dennison, M. S., Sivaram, N. M., Barik, D. & Ponnusamy, S. Turning operation of AISI 4340 steel in flooded, near-dry and dry conditions: A comparative study on tool-work interface temperature. *Mech. Mech. Eng.* **23**, 172–182. 10.2478/mme-2019-0023 (2019).

[54] Islam, M. T. T., Islam, M. T. T., Chowdhury, S. A., Mourshed, M. & Masud, M. H. A comparative numerical analysis of heat generation pattern for different materials at varying cutting speed. *AIP Conf. Proc.*10.1063/1.5115958 (2019).

[55] Yanda, H. Effect of cutting speed on cutting force and temperature in turning process. *J. Inov. Rekayasa Mek. Dan Termal* **1**, 46–52. 10.25077/inomet.1.1.46-52.2023 (2023).

[56] Senthilkumar, P., Senthilkumar, R. & Marichelvam, B. Effect of feed rate on material removal rate, surface roughness and machining time of aluminum alloy 6063 in CNC turning. *Int. J. Sci. Res. Sci. Eng. Technol.* **4099**, 142–147. 10.32628/ijsrset229518 (2022).

[57] Latif, A. A., Ibrahim, M. R., Amran, A. Z. & Rahim, E. A. A study on the effect of feed rate and cutting speed on surface roughness and material removal rate of mild steel. *IOP Conf. Ser. Mater. Sci. Eng.*10.1088/1757-899X/257/1/012025 (2017).

[58] Cui, X., Zhao, B., Jiao, F. & Zheng, J. Chip formation and its effects on cutting force, tool temperature, tool stress, and cutting edge wear in high- and ultra-high-speed milling. *Int. J. Adv. Manuf. Technol.* **83**, 55–65. 10.1007/s00170-015-7539-7 (2016).

[59] Haghnazari, S. & Abedini, V. Effects of hybrid Al2O3–CuO nanofluids on surface roughness and machining forces during turning AISI 4340. *SN Appl. Sci.*10.1007/s42452-020-04088-w (2021).

[60] Dennison, M. S., Jebabalan, S. K. & Barik, D. Applicability of nano-cutting fluids for enhanced cooling, low tool wear, and high tribological performance during machining—a review. *Discov. Appl. Sci.*10.1007/s42452-024-06398-9 (2024).

[61] Gupta, A., Kumar, R., Kumar, H., Mehta, J. S. & Wadhwa, A. S. Experimental study on the effect of combination ratio of Al2O3-MWCNT hybrid nano-cutting fluids while turning AISI 304. *Eng. Res. Express*10.1088/2631-8695/ad9b01 (2024).

[62] Veerappan, G. et al. Experimental and numerical analysis on the cutting force, cutting temperature, and tool wear of alloy steel (4340) during turning process. *AIP Adv.*10.1063/5.0227710 (2024).

[63] Liang, S. Y. & Shih, A. J. Cutting temperature and thermal analysis. *Anal. Mach. Mach. Tools*10.1007/978-1-4899-7645-1_9 (2016).

[64] Uwabuike, V. C., Nwufo, O. C., Azubuike, J. O. & Nwaji, G. N. Experimental investigation of temperature distribution in the chips, workpiece and cutting tool during machining operations. *J. Appl. Phys. Sci. Int.* **15**, 1–23. 10.56557/japsi/2023/v15i28370 (2023).

[65] Ye, G. G. et al. Cutting AISI 1045 steel at very high speeds. *Int. J. Mach. Tools Manuf.* **56**, 1–9. 10.1016/j.ijmachtools.2011.12.009 (2012).

[66] Jiang, H. et al. Influence of cutting velocity on gradient microstructure of machined surface during turning of high-strength alloy steel. *Mater. Sci. Eng. A*10.1016/j.msea.2021.141354 (2021).

[67] Mandal, N., Doloi, B. & Mondal, B. Predictive modeling of surface roughness in high speed machining of AISI 4340 steel using yttria stabilized zirconia toughened alumina turning insert. *Int. J. Refract. Met. Hard Mater.* **38**, 40–46. 10.1016/j.ijrmhm.2012.12.007 (2013).

[68] Sutter, G. & List, G. Very high speed cutting of Ti-6Al-4V titanium alloy - Change in morphology and mechanism of chip formation. *Int. J. Mach. Tools Manuf.* **66**, 37–43. 10.1016/j.ijmachtools.2012.11.004 (2013).

[69] Sahoo, P. & Patra, K. Influences of feed rate and machining length in micro-milling of P-20 steel. *Lect. Notes Mech. Eng.*10.1007/978-981-15-1307-7_13 (2020).

[70] Naghizadeh, H. et al. Effect of using CuO–oil nanofluid on surface roughness and thermal performance during superfinishing process. *Adv. Eng. Mater.*10.1002/adem.202201918 (2023).

[71] Bai, X. et al. Tribological performance of different concentrations of Al2O3 nanofluids on minimum quantity lubrication milling. *Chinese J. Mech. Eng.*10.1186/s10033-022-00830-0 (2023).

[72] Jamil, M., He, N., Zhao, W., Khan, A. M. & Laghari, R. A. Tribology and machinability performance of hybrid Al2O3 -MWCNTs nanofluids-assisted MQL for milling Ti-6Al-4 V. *Int. J. Adv. Manuf. Technol.* **119**, 2127–2144. 10.1007/s00170-021-08279-6 (2022).

[73] Basavarajappa, S., Suresh, R., Gaitonde, V. N. & Samuel, G. L. Analysis of cutting forces and surface roughness in hard turning of AISI 4340 using multilayer coated carbide tool. *Int. J. Mach. Mach. Mater.*10.1504/IJMMM.2014.064687 (2014).

[74] Liang, X., Liu, Z., Wang, B., Wang, C. & Cheung, C. F. Friction behaviors in the metal cutting process: state of the art and future perspectives. *Int. J. Extrem. Manuf.*10.1088/2631-7990/ac9e27 (2023).

[75] Dogra, M. & Sharma, V. S. Machinability and surface quality issues in finish turning of hardened steel with coated carbide and CBN tools. *Mater. Manuf. Process.* **27**, 1110–1117. 10.1080/10426914.2011.654164 (2012).

[76] Karpat, Y. & Özel, T. Analytical and thermal modeling of high-speed machining with chamfered tools. *J. Manuf. Sci. Eng.* **130**, 0110011–01100115. 10.1115/1.2783282 (2008).

[77] Shih, Y. P., Wang, Y. C., Wei, B. L. & Chen, K. H. Material removal rate of face-milled bevel gears based on a ring-dexel cutting simulation. *J. Manuf. Sci. Eng.*10.1115/1.4066798 (2025).

[78] Amigo, F. J. et al. On the effects of cutting-edge angle on high-feed turning of Inconel 718© superalloy. *Int. J. Adv. Manuf. Technol.* **125**, 4237–4252. 10.1007/s00170-023-10974-5 (2023).

[79] Mehmood, T. & Khalil, M. S. Enhancement of machining performance of Ti-6Al-4V alloy though nanoparticle-based minimum quantity lubrication: insights into surface roughness, material removal rate, temperature, and tool wear. *J. Manuf. Mater. Process.*10.3390/jmmp8060293 (2024).

[80] Kamble, P. D., Sahare, S. B., Untawale, S. P. & Mehta, H. D. Taguchi design of experiments to optimize material removal rate in turning AISI 4340 steel. *J. Phys. Conf. Ser.*10.1088/1742-6596/2763/1/012013 (2024).

[81] Kataoka, R. & Shamoto, E. Influence of vibration in cutting on tool flank wear: Fundamental study by conducting a cutting experiment with forced vibration in the depth-of-cut direction. *Precis. Eng.* **55**, 322–329. 10.1016/j.precisioneng.2018.09.021 (2019).

[82] Paturi, U. M. R. et al. Estimation of surface roughness of direct metal laser sintered AlSi10Mg using artificial neural networks and response surface methodology. *Mater. Manuf. Process.* **38**, 1798–1808. 10.1080/10426914.2023.2217890 (2023).

[83] Al-Ani, H. H. H. Artificial neural network in the prediction of surface roughness: A comparative study. *Sustain. Eng. Innov.* **5**, 141–150. 10.37868/sei.v5i2.id216 (2023).

[84] Nguyen, V. H., Le, T. T. & Nguyen, A. T. Prediction model for surface roughness of polycarbonate using single-point-diamond-turning lathe machining based on machine learning techniques. *Lect. Notes Mech. Eng.*10.1007/978-3-031-39090-6_23 (2024).

[85] Nguyen, V. H., Le, T. T., Nguyen, A. T., Hoang, X. T. & Nguyen, N. T. Multiobjective optimization of end milling parameters for enhanced machining performance on 42CrMo4 using machine learning and NSGA-III. *Mach. Sci. Technol.* **28**, 744–776. 10.1080/10910344.2024.2381191 (2024).

[86] Okokpujie, I.P., Azeez, T.M., Lawan, R.O., Agbemuko, D.I., Okokpujie, K., Omoyi, C.O. Significant effects of nano-lubrication mechanism on milling process: An overview. In *2024 IEEE 5th International Conference on Electro-Computing Technologies for Humanity (NIGERCON)* (2024). 10.1109/NIGERCON62786.2024.10927378.

[87] Unune, D. R., Nirala, C. K. & Mali, H. S. ANN-NSGA-II dual approach for modeling and optimization in abrasive mixed electro discharge diamond grinding of Monel K-500. *Eng. Sci. Technol. an Int. J.* **21**, 322–329. 10.1016/j.jestch.2018.04.014 (2018).

[88] Tzotzis, A., Nedelcu, D., Mazurchevici, S. N. & Kyratsis, P. Surface quality evaluation of 3d-printed carbon-fiber-reinforced PETG polymer during turning: experimental analysis, ANN modeling and optimization. *Polymers (Basel).*10.3390/polym16202927 (2024). 39458759 10.3390/polym16202927 PMC11511286

